# Intrinsic disorder in protein domains contributes to both organism complexity and clade-specific functions

**DOI:** 10.1038/s41598-021-82656-9

**Published:** 2021-02-04

**Authors:** Chao Gao, Chong Ma, Huqiang Wang, Haolin Zhong, Jiayin Zang, Rugang Zhong, Fuchu He, Dong Yang

**Affiliations:** 1 State Key Laboratory of Proteomics, National Center for Protein Sciences (Beijing), Beijing Proteome Research Center, Beijing Institute of Lifeomics, 38 Science Park Road, Changping District, Beijing, 102206 China; 2 Beijing Key Laboratory of Environmental and Viral Oncology, College of Life Science and Bioengineering, Beijing University of Technology, Beijing, 100124 China

**Keywords:** Evolution, Protein sequence analyses

## Abstract

Interestingly, some protein domains are intrinsically disordered (abbreviated as IDD), and the disorder degree of same domains may differ in different contexts. However, the evolutionary causes and biological significance of these phenomena are unclear. Here, we address these issues by genome-wide analyses of the evolutionary and functional features of IDDs in 1,870 species across the three superkingdoms. As the result, there is a significant positive correlation between the proportion of IDDs and organism complexity with some interesting exceptions. These phenomena may be due to the high disorder of clade-specific domains and the different disorder degrees of the domains shared in different clades. The functions of IDDs are clade-specific and the higher proportion of post-translational modification sites may contribute to their complex functions. Compared with metazoans, fungi have more IDDs with a consecutive disorder region but a low disorder ratio, which reflects their different functional requirements. As for disorder variation, it’s greater for domains among different proteins than those within the same proteins. Some clade-specific ‘no-variation’ or ‘high-variation’ domains are involved in clade-specific functions. In sum, intrinsic domain disorder is related to both the organism complexity and clade-specific functions. These results deepen the understanding of the evolution and function of IDDs.

## Introduction

Proteins containing intrinsically disordered regions (IDR)^1,2^ are called intrinsically disordered proteins (IDP, also named as intrinsically unstructured^3,4^ or natively unfolded proteins^5^), which haven no stable ordered structure but have important biological functions via interacting with various partners^1–3,6^. According to our empirical knowledge, protein domains, as conserved units that can evolve, fold^7^ and function^8^ independently, are the structured and compact regions^9^ in contrast to IDRs. However, some protein domains indeed can exist and function without a stable three-dimensional structure. This type of protein domains was named as intrinsically disordered domains (IDDs). During the last two decades, an increasing number of IDDs have been reported^10–14^.

Previous studies mainly focused on the special physical–chemical properties and functional characteristics of certain IDDs. For example, Dogan *et al.*^15^ found that the binding rates of disordered domains are faster than those of structured domains. Stanley *et al.*^16^ showed that in the cAMP response element binding protein (CREB), the disordered kinase inducible domain (KID) could become structured after its phosphorylation. Furthermore, a research has revealed that IDDs can participate in the formation of dynamic protein complexes and prevent additional protein monomer polymerization to maintain protein stability^17^.

The preceding studies mainly focused on different individual disordered protein domains. With the advancement of genome sequencing of human and model organisms, the characteristics of IDDs at the entire proteome level have been explored by some studies. Chen *et al.* explored the distribution pattern of the conserved predicted disordered regions in domains and domain families^18^, and this kind of IDDs can mediate protein–protein recognition^19^. However, the distribution pattern of the IDDs across the species of the three superkingdoms and its functional significance are unclear. To this end, the calculation of the number of IDDs encoded by a genome and a comprehensive exploration of their evolutionary and functional features are important; such research will enrich our understanding of the evolution of the protein repertoire.

More specifically, this analysis will also provide valuable insights into the mechanism of the increasing organismal complexity during evolution. Previous studies suggest hub proteins usually have higher content of intrinsic disorder to interact with various partners and form complex networks^6,20–25^ and IDPs substantially contribute to the increasing organism complexity^26,27^. It is known clearly that eukaryotes have more proportion of intrinsically disordered proteins than prokaryotes^26–31^. However, the proportion of disorder at protein level in eukaryotes has no good correlation with species complexity^26,27^. Here, we investigated the relationship between the proportion of intrinsic disorder at domain level and eukaryotic organism complexity.

In this study, there are two measures of the degree of disorder: the domain structural disorder ratio (DSDR), represented by the percentage of disordered residues in one domain; and CDRN, the number of consecutive disordered regions (CDR). CDRs are the regions where the consecutive disordered residues are more than 20 in the domains longer than 50 residues, or more than 40% of the total residues in the other domains. The relationship between these two measures had not been explored before. We investigated their relationship and compared the relationship patterns among different evolutionary lineages.

Protein domains usually repeat in the same proteins or are shared among different proteins. The unique protein domain types are defined as domain families^32,33^. Each domain family has a unique domain identifier and name (See Method section for its detailed definition). Based on usual understanding, the protein domains belonging to a same family should have the same structural characteristics. To test whether this intuitive understanding is correct, the variation of the disorder degree of the same protein domains was investigated in our analysis. Furthermore, we investigated the factors that affect the variation of domain disorder and the biological significance of their variation.

## Results

### Distribution of IDDs across the three superkingdoms

**Figure 1:**
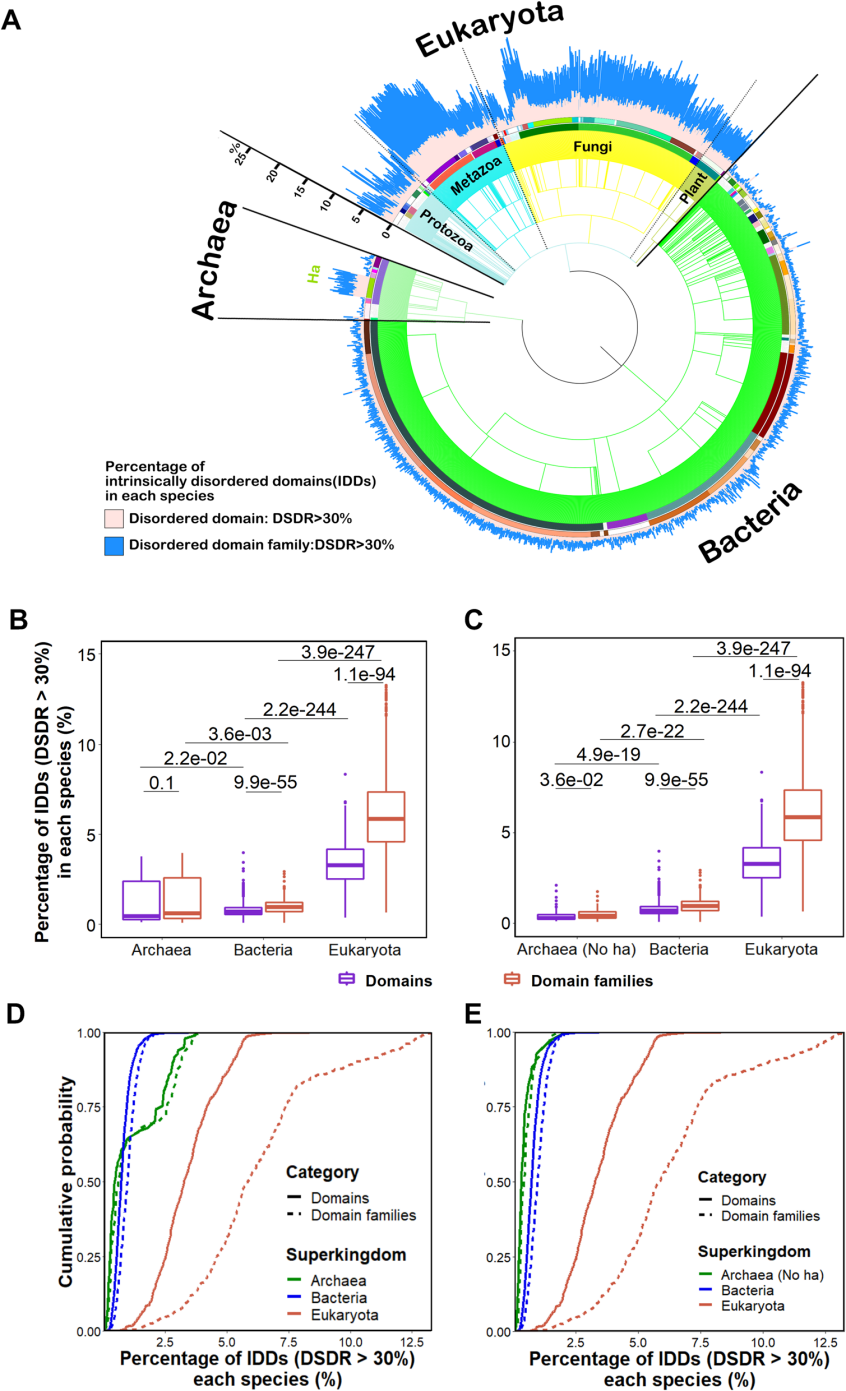
Distribution of IDDs in 1870 species across the three superkingdoms. (**A**) The percentage of IDDs (DSDR > 30%) in each species from archaea (light green), bacteria (lime) and eukaryotes. Each bar indicates the results of a species. All species are arranged according to taxonomy information from NCBI database. The superkingdoms are separated by solid lines and the kingdoms in eukaryotes, including protists (light blue)**,** metazoa (aqua), fungi (yellow) and plant (pale goldenrod), are separated by dashed lines. Each phylum is filled with different colors in the middle circle. The outer circle represents different classes. The detailed information about the names and colors of each species, class and phylum is presented in supplementary table S1–S3. The abbreviations in this figure: Ha, the class of Halobacteria; DSDR, domain structural disorder ratio. (**B**) Box-plot of the percentage of intrinsically disordered (DSDR > 30%) domain (purple) or domain families (red) in each species across the three superkingdoms (**C**, excluding halobacteria). For box-plots, the values of upper and lower quartile are indicated as upper and lower edges of the box, and the median values are indicated as a bar in the box. The differences in percentage of disordered domain distribution between different categories are examined by Mann–Whitney U test. The corrected *P* values are shown at the top of each panel. (**D**) Cumulative probability of the percentage of intrinsically disordered domain (solid line) or domain families (dashed line) in archaea (blue), bacteria (green) and eukaryotes (red) (**E**, excluding halobacteria).

In order to achieve a complete representation of the distribution pattern of IDDs across the three superkingdoms, one representative species was chosen from each genus. In total, 1,870 species were involved in the calculation and analysis of domain disorder, including 97 species of archaea, 1224 species of bacteria and 549 species of eukaryotes (Dataset 1, 2; including 135 metazoans, 304 fungi, 65 protists, and 45 plants). We found that the percentages of IDDs (DSDR > 30%, or CDRN ≥ 1) are highest in eukaryotes compared with the other two superkingdoms (Fig. 1, S1, and table S1A, S2A for the results based on SPOTD; Table S1B, S2B for the results based on IUPred; Figure S2 and table S1C, S2C for the results based on MobiDB-lite). From the viewpoint of domain families, the degree of disorder of each domain family is defined by the dominant category (Figure S3). The median proportion of IDD families in eukaryotes is 10.1% (See table S2A for raw data), while in archaea and bacteria the corresponding value is 1.5% (See table S2A for raw data). These results indicate that evolutionarily more complex species (hereinafter, abbreviated as ‘complex species’) have a considerably higher proportion of IDDs.

One of the most obvious exceptions to this observation is found in archaeal halobacteria, which contain an even higher proportion of IDDs than eukaryotes (Fig. 1A). When halobacteria were excluded from archaea, the ratio of IDDs per species in archaea was the lowest among the three superkingdoms (Fig. 1B–E, S1B-E, S2A–D). Other studies have also found that halobacteria have an unusually high ratio of disordered proteins^34^. However, another literature reported that this phenomenon was due to methodological bias^35^. Our results show that the original conclusion is valid even if the method is corrected (Figure S4). The main innovation of the current study is that we focused on disorder at the domain level, while previous studies focused on the disorder at protein level.

Comparing the percentage of IDDs calculated at domain level (Table S1) to that at domain family level (Table S2), we found an interesting phenomenon: in eukaryotes, the percentages of IDDs were considerably lower than those of the disordered domain families (Fig. 1B–E, S1B–E, S2A–D), whereas there are no such significant differences between domain and domain families in archaea and bacteria. This phenomenon may be related to the number of domain repeats in eukaryotes. The average number of repeats of the disordered domain families was obviously less than that of the completely structured domain families (Figure S5, Table S4). Completely structured domains in eukaryotes tend to repeat many times.

The above results revealed that there is an obvious difference between prokaryotes and eukaryotes in the IDD distribution pattern. We next focused on the difference of IDD distribution of different clades within eukaryotes.

### The correlation between intrinsic disorder of protein domain and eukaryotic organism complexity

**Figure 2:**
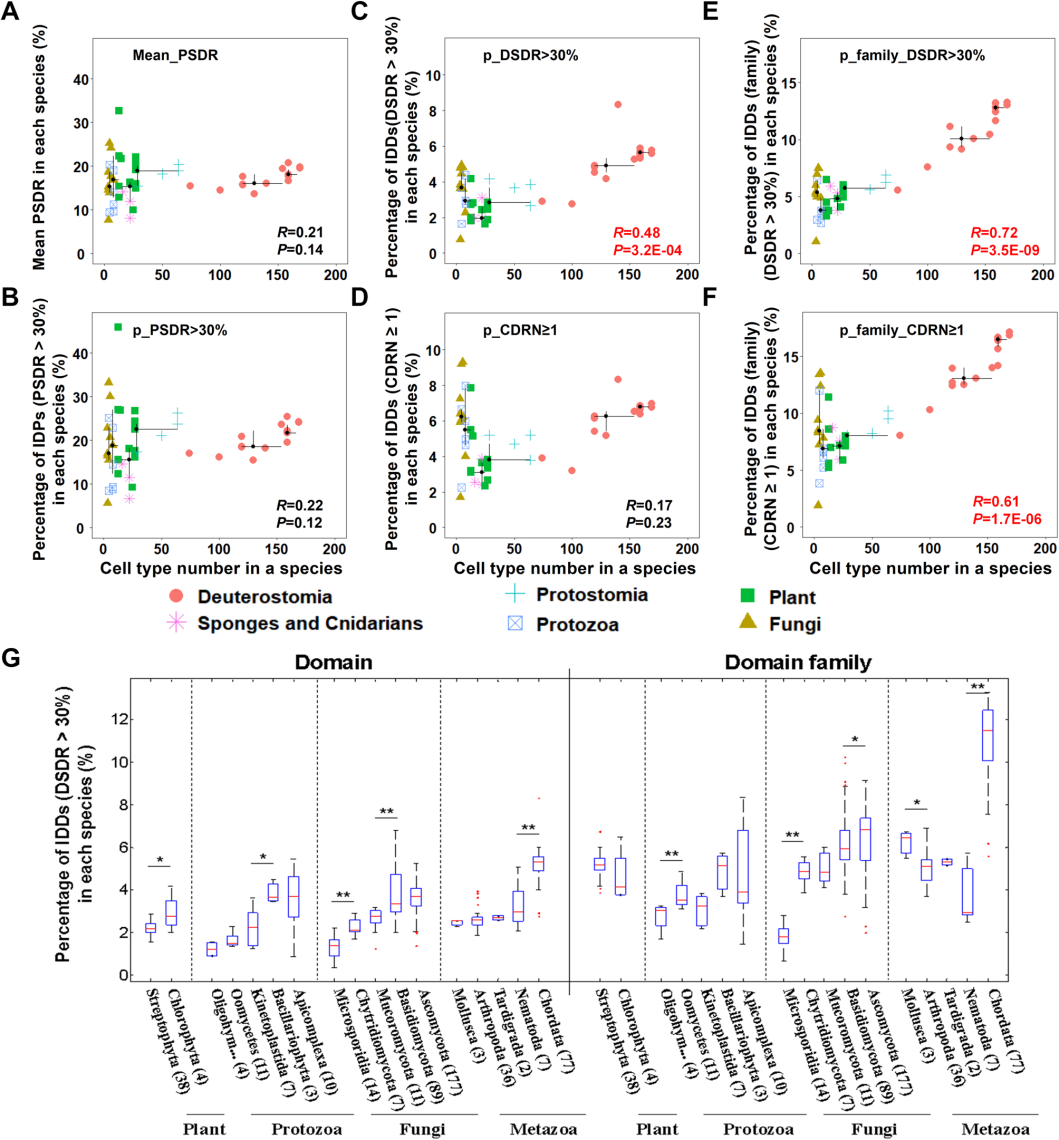
The correlation between structural disorder and organism complexity. (**A**) Protein disorder degree was measured by the average of all representative proteins (the longest one for each gene) in each species. (**B**) Proteins with more than 30% disordered amino acids (PSDR > 30%) were regarded as disordered proteins. Protein disorder degree was measured by the percentage of disordered proteins in each species. (**C, D**) At domain level, structural disorder of each species was measured by the percentage of IDDs (C, DSDR > 30%, or D, CDRN ≥ 1). (**E, F**) At domain family level, domain families were defined using dominant category method; structural disorder of each species was measured by the percentage of intrinsically disordered domain families (**E**, family_DSDR > 30%, or F, family_CDRN ≥ 1). Scatter plots was made for organism complexity and structural disorder values obtained by different methods in fifty-one eukaryotes. Spearman method was used for the correlation analysis. The correlation coefficients (R) and *P* values (*P*) are shown in the inset, among which the significant results are shown as red. (**G**) Box-plot of the percentage of intrinsically disordered (DSDR > 30%) domains (left panel) or domain families (right panel) in each species across the different phyla of eukaryotes. The four kingdoms of eukaryotes are marked on the bottom of the figure. The values of upper and lower quartile, and the median values are indicated as bars in the boxes. The differences in percentage of disordered domain distribution between adjacent categories are examined by Mann–Whitney U test. The corrected *P* values are marked as stars in sub-figure G (*P  < 0.05; ** P  < 0.01).

Different eukaryotes have great differences in complexity. Some are only unicellular eukaryotes, while others have hundreds of cell types. The organism complexity of eukaryotes can be measured by the number of cell types in this species^36–40^. In previous studies, Schad *et al.*^26^ and Xue *et al.*^27^ reported that the fraction of protein structural disorder increases significantly between prokaryotes and eukaryotes but within eukaryotes there was no significant correlation between organism complexity and the average disorder content in proteins. We confirmed that protein disorder does not significantly correlate well with organism complexity, whether we use the average value of protein structural disorder ratio (PSDR, Fig. 2A, S6A) or the percentage of disordered proteins (PSDR > 30%) (Fig. 2B, S6B) to represent the disorder degree of proteome. However, there is a significant positive correlation between the percentage of disordered protein domain (DSDR > 30%) and organism complexity (Fig. 2C, S6C), but if CDRN was used to define disordered protein domain, there is no significant correlation (Fig. 2D, S6D), indicating that in complex species there are more disordered domains with higher ratios of disordered residues, not with a consecutive disordered region. These results revealed that compared with the simple eukaryotes, the complex ones contain much more IDDs, but at protein level, they have no such obvious difference in the proportion of intrinsically disordered proteins (IDPs). This implies that IDDs may contribute to the complex functions in evolutionarily complex eukaryotes.

When domain repetition of the same domain family in one species was not taken into account, a stronger correlation can be obtained (Fig. 2E–F, S6E–F), suggesting complex eukaryotes have more IDDs without repetition compared with simple eukaryotes. This means factors unrelated to organism complexity are added when considering domain repetitions (Fig. 2C–D, S6C–D). The proportion of IDDs in complex eukaryotes will decrease when the domain repetitions are included, because structured domains have more repetition counts than disordered domains.

These results suggest that domain disorder, rather than protein disorder, is an important factor contributing to eukaryotic organism complexity. Intrinsic disorder in protein domains may be an important factor to promote the increasing complexity of eukaryotes.

However, the comparison of different species of eukaryotes showed that the percentage of IDDs in simpler organisms is even higher than that of complex organisms (Fig. 2G, Figure S7). For example, the percentages of IDDs in ascomycetes or basidiomycetes, two phyla of fungi, are much higher than those in some metazoans, such as arthropods. However, according to the cell type number of an organism (Approximately: 5 for these two phyla of fungi *vs.* 64 for arthropod *Drosophila melanogaster*)^26,37^, the organismal complexity of arthropods is much higher than fungi of these two phyla. Thus, intrinsic domain disorder in a species is not only related to organismal complexity, but may also be related to special functions, which will be discussed in the following text (Part 4 results).

### The causes of uneven distribution of IDDs among species

The preceding results show that, in general, the percentage of IDDs in species is positively related to organism complexity, that is, there is a greater proportion of IDDs in complex species; but in some instances, there are also more IDDs in some simpler species compared to complex species. What is the reason for the uneven distribution of the proportion of IDDs across species? This may be related to two aspects of protein domain evolution: firstly, some clade-specific domains (only appear in the species of a certain evolutionary clade) may tend to be more disordered, leading to the higher proportion of IDDs in these species, especially in complex eukaryotic species; secondly, the degree of disorder of some common domains (appear widely across a kingdom, even across superkingdoms) diverged during evolution, that is, some domains from the same families may have remarkably different DSDR values in different species.

**Figure 3:**
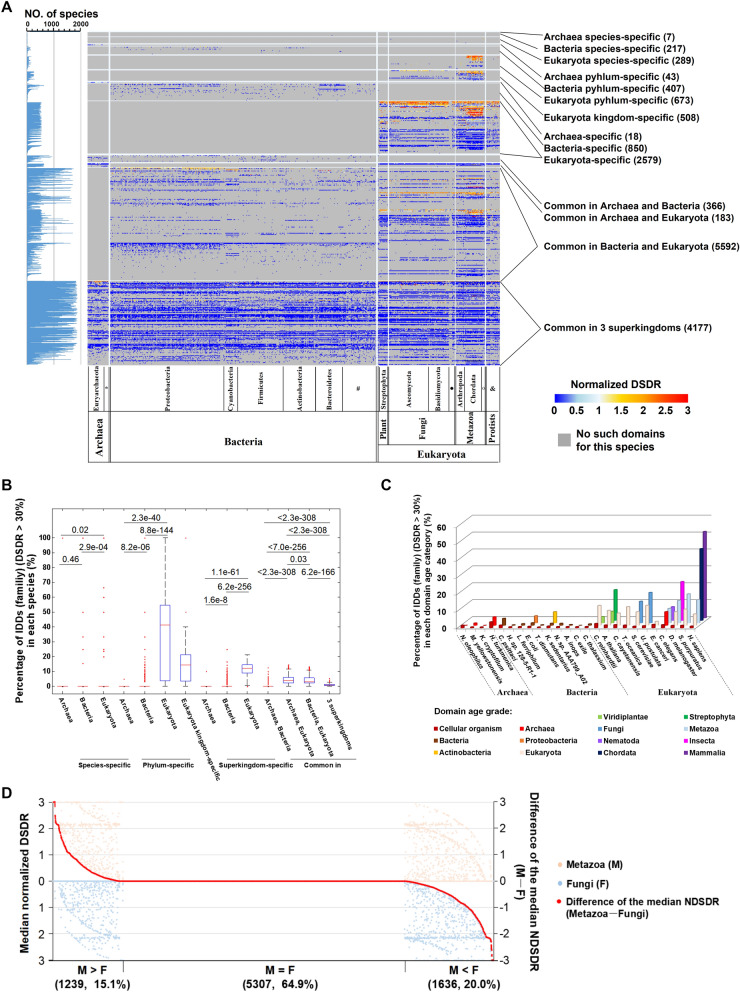
The correlation between domain age and domain disorder degree. (**A**) Heat map of the DSDR distribution of all protein domains in different species across three superkingdoms. The normalized DSDR (see method) of the same domain family within one species is represented by the median value. The number of species containing a certain domain family is shown on the left panel. Domain families are classified according to their distribution width (right panel). The numbers of domain families in each category are shown in brackets. The taxonomic names of the phylum, kingdom and superkingdom for each species are marked on the bottom of the figure. The symbols representing multiple taxonomic names in this figure: *Candidatus Korarchaeota, Crenarchaeota and Thaumarchaeota; ^#^Fibrobacteres, Aquificae, Chlamydiae, Chloroflexi, Fusobacteria, Acidobacteria, Planctomycetes, Chlorobi, Thermodesulfobacteria, Coprothermobacterota, Dictyoglomi, Thermotogae, Lentisphaerae, Verrucomicrobia, Synergistetes, Gemmatimonadetes, Elusimicrobia, Armatimonadetes, Caldiserica, Deferribacteres, Spirochaetes, Chrysiogenetes, Calditrichaeota, Ignavibacteriae, Candidatus Saccharibacteria, Candidatus Omnitrophica, Nitrospinae, Candidatus Peregrinibacteria, Balneolaeota, Candidatus Tectomicrobia; filled circle, Microsporidia, Chytridiomycota, Blastocladiomycota, Zoopagomycota, Cryptomycota; open circle, Placozoa, Ctenophora, Mollusca, Cnidaria, Tardigrada, Porifera. (**B**) Box-plot of the percentage of intrinsically disordered (DSDR > 30%) domain families in each category of domain distribution width. The four kingdoms of eukaryotes are marked on the bottom of the figure. The values of upper and lower quartile, and the median values are indicated as bars in the boxes. The differences between different categories are examined by Mann–Whitney U test. The corrected *P* values are shown. (**C**) The distribution of IDDs (DSDR > 30%) in each age grade of the 25 representative species from archaea, bacteria and eukaryotes. Domains of each representative species are divided into different age grades. (**D**) Comparison of the normalized DSDR values of the same domain families in metazoa and fungi. Scatter-plot shows the median normalized DSDR values of the same domain families in metazoa (top half of the figure) and fungi (bottom half of the figure). The red dots represent the difference of the median normalized DSDR value of each domain family between metazoa and fungi. The number and percentage of domain families belonging to each differential category are shown in brackets.

In order to test these two hypotheses, we first classified protein domains into 14 categories according to the species distribution width (Fig. 3A, Dataset 3). For the first hypothesis, the percentage of IDDs in each category was calculated. It was clear that eukaryote-specific domains, including species-specific, phylum-specific, kingdom-specific and superkingdom-specific domains, contained a higher proportion of IDDs than prokaryote-specific domains (Fig. 3B). The distribution width of a domain could be used as an indicator of evolutionary origin time (domain age); thus, we can infer that young domains (clade-specific domains) tend to be IDDs especially in eukaryotes. As examples, the data of 25 representative species were used to calculate the proportions of IDDs in the domains with different domain age grades. We found that younger domains consist of a higher fraction of IDDs than older ones, particularly in complex eukaryotes (Fig. 3C, S8A–C). For example, in humans, over half of the mammalian-specific domains are IDDs.

For the second hypothesis, we compared the degree of disorder of the same domains among different species. For the domains common in metazoans and fungi, 15.1% of them had higher degrees of disorder in metazoans, whereas 20.0% of them had higher degrees of disorder in fungi (Fig. 3D). Thus, the degree of disorder of old domains (widely distributed domains) varies considerably among species, but the degree of disorder is not always high in complex species. The comparisons between eukaryotes and prokaryotes (Figure S9A), multicellular eukaryotes and unicellular eukaryotes (Figure S9B), metazoan and protozoa (Figure S9C), and between chordates and other metazoans (Figure S9D) also supported this conclusion.

These two factors led to the fact that each evolutionary clade may have its own specific young IDDs and special ancient IDDs.

### The biological functions of IDDs

**Figure 4:**
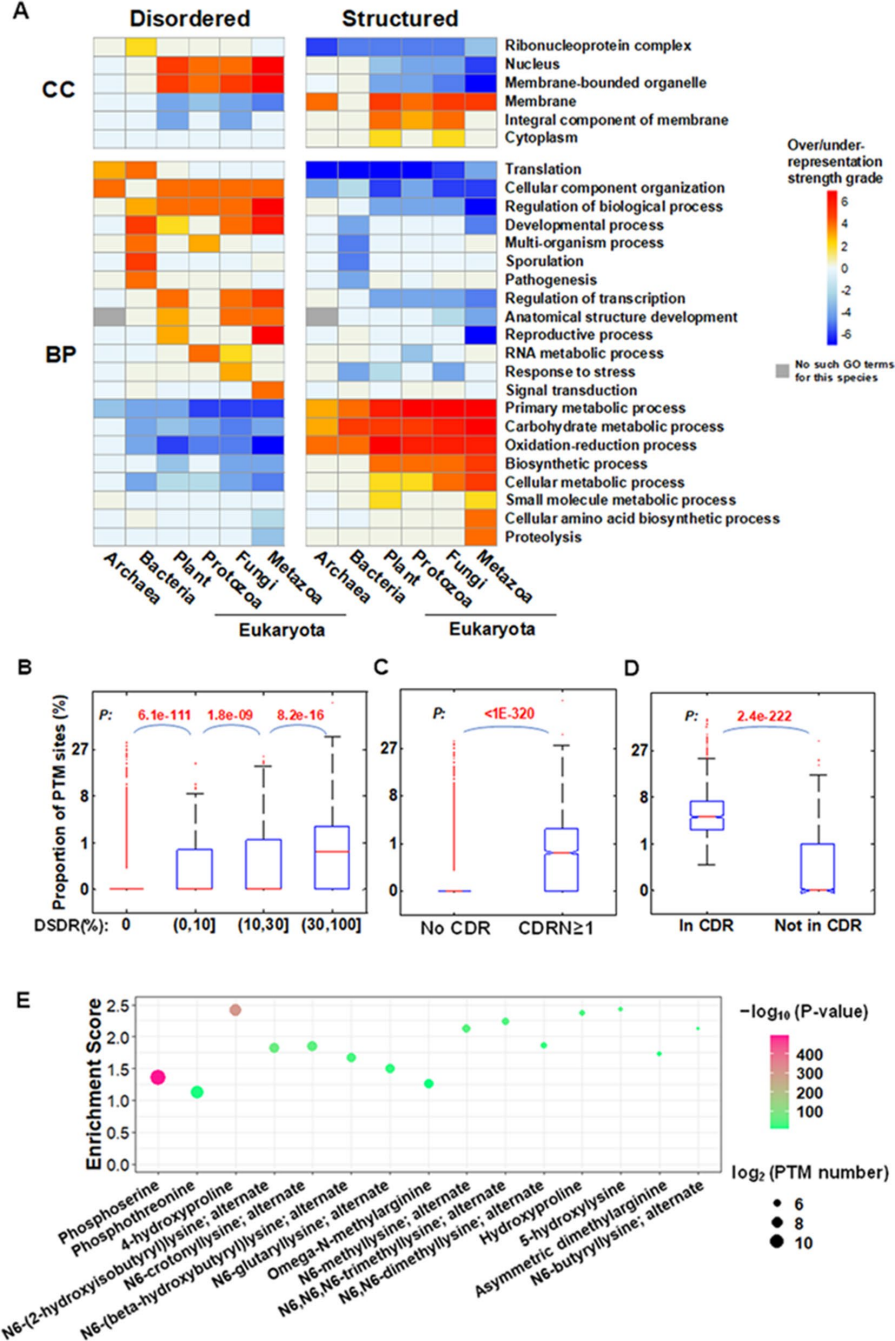
Functional features of intrinsically disordered domains. (**A**) Over- or under-representation analysis of biological process (BP) and cellular component (CC) for the IDDs in archaea, bacteria and eukaryotes. The over- or under-representation strengths of each class were represented by − log (*P*) or log (*P*), respectively. Heat map showing the grades of over- or under-representation strengths, scoped from − 7 to 7. (**B**–**D**) Comparison of the PTM site proportion in different type of domains/regions classified according to disorder degree. Box-plot of the PTM site proportion in protein domains with different disorder degree classified according to DSDR (**B**) or CDRN (**C**), or in CDR regions or other regions in domains (**D**). The values of upper and lower quartile, and the median values are indicated as bars in the boxes. The differences between the neighboring categories are examined by Mann–Whitney U test. The corrected *P* values are shown. (**E**) The significantly over-represented PTM types in IDDs. The over-representation strengths of each PTM type were represented by − log (*P*), marked with different colors. The number of each type of PTM sites in IDDs were indicated as the size of the circles. The analyses about PTM in disordered domains/regions were based on human data (**B**–**E**). The results of other six species were in figure S11 and table S5.

As domains are the functional units in proteins, we assume that the domains with different degrees of disorder have different biological functions. In order to test this hypothesis, all of the IDDs (DSDR > 30%, or CDRN ≥ 1 in no less than 50% species of each group) and the completely structured domains (DSDR = 0 in no less than 50% species of each group) were subjected to the over-representation analysis of gene ontology (GO). We found that the completely structured domains tend to localize at membranes, and participate in the biological processes related to metabolism, including various biosynthetic and catabolic processes, as well as oxidation–reduction process (Fig. 4A). In contrast, the IDDs in prokaryotes tend to localize at ribonucleoprotein complex, and tend to participate in translation and cellular component organization. The IDDs in bacteria are also specifically related to pathogenesis and sporulation. In eukaryotes, the IDDs are more likely to localize at the nucleus and participate in different regulatory processes. For example, in most cases, the basic leucine zipper domain (pfam00170) is an IDD, which exists in many eukaryotic DNA binding proteins. Most of the proteins containing this domain are transcription factors. In addition, some kingdom-specifically over-represented terms can be observed. For example, ‘signal transduction’ is specifically over-represented in metazoan IDDs; ‘response to stress’ is specifically over-represented in fungi IDDs. These results revealed that the IDDs in different evolutionary clades tend to perform different types of biological functions.

More specifically, we investigated the special functional characteristics of IDDs in archaeal halobacteria (Figure S10) and two phyla of fungi (Figure S11). Compared with other archaebacteria, 380 IDDs (82%) in halobacteria are halobacteria-specific (Figure S10A). Halobacteria-specific IDDs tend to be involved in specific metabolic processes, such as the metabolism of amino acid, oxoacid, and oxidation–reduction process (Figure S10B,C). The participation of halobacteria-specific IDDs in these specific metabolic processes may be the requirements of their special halophilic ability. To investigate the fungi-specific functions related to IDDs, we acquired protein phenotype dataset from Pathogen-Host Interaction (PHI) database^41^ (http://www.phi-base.org), which includes over 7000 proteins from over 200 species with phenotype annotation based on mutation experiment, and we explored the specific mutant phenotypes related to the proteins with IDDs in ascomycetes and basidiomycetes (two phyla of fungi), compared with other species. As the result, the proteins with IDDs of these two phyla of fungi significantly enriched in proteins whose mutation would lead to ‘reduced virulence’ compared with non-IDD domains (Figure S11). In contrast, proteins with IDDs of bacteria or parasites are not enriched in this phenotype. This result indicates that IDDs in ascomycetes and basidiomycetes are related to their virulence and fitness.

As mentioned in Fig. 3, protein domains could be classified into different categories according to species distribution width (domain age). Since the same protein domains may have different degrees of disorder in different species, we also calculated the percentage of species where the domain is an IDD (disordered width). Based on these two parameters, domain age and the disordered width, all the domains were divided into six categories (Figure S12). For the old domains, appearing in both eukaryotes and prokaryotes, the disordered width has a great influence on the functional type. The widely disordered old domains (disordered in more than 50% species) mainly participate in the regulatory processes, whereas the old domains, which are disordered in less than 50% species are mainly involved in metabolic processes, including oxidation–reduction, biosynthetic and catabolic processes, similar to the enriched biological processes for completely structured domains in Fig. 4A. The eukaryote-specific or prokaryote-specific domains both have their specifically enriched GO terms. For example, eukaryote-specific domains tend to participate in regulation of transcription, reproductive and developmental processes, signal transduction, and intracellular transport, etc. For example, Androgen_recep domain (androgen receptor, PF02166, Fig S13A) is a young (chordates-specific) and widely disordered domain, which is found in 54 chordate species involved in this study. All of the Androgen_recep domains in 42 mammals are highly disordered (DSDR > 30%). In human, this domain, with a high degree of disorder (DSDR = 63.5%, CDRN = 2), exists in the androgen receptor protein (AR, P10275), which can regulate gene expression and affect cellular proliferation and differentiation in target tissues. Prokaryote-specific domains tend to specifically participate in pathogenesis, interspecies interactions (for both types of disordered width), and DNA modification, the generation of precursor metabolites and energy (for the IDDs in less than 50% species). The examples for each of the six categories of IDDs are shown in figure S13. These results reveal that the functions of IDDs are related to both domain age and disordered width, and disordered width has a greater influence on the functions of old IDDs, while the functions of young IDDs are mainly related to their clade-specificity.

Post-translational modifications (PTM) usually play important roles in protein–protein interaction and regulation of the activities of most of the protein machines in cells. Previous studies revealed IDRs are frequently subjected to PTMs that increase the functional states^1,31,42,43^. Thus, we speculate that IDDs may provide more residues to PTM than structured domains. We tested this hypothesis based on 7 representative species. It’s obvious that there are significant differences in proportion of PTM sites between structured and disordered protein domains (Fig. 4 B–D, Figure S14). The proportion of PTM sites is much higher in the intrinsically disordered protein domains [DSDR > 30% (Fig. 4B, Figure S14A), or CDRN ≥ 1 (Fig. 4C, Figure S14B)] than the structured domains (DSDR = 0, or CDRN = 0). Within the IDDs with CDRs, the CDRs have much higher proportion of PTM sites than other regions of these IDDs (Fig. 4D, Figure S14C). The significantly over-represented PTM types in intrinsically disordered domains were explored (Fig. 4E, Table S5). Phosphorylation of serine and threonine, acetylation, methylation and crotonylation of lysine, hydroxylation of proline and lysine, methylation of arginine etc*.* are the most over-represented PTM types in IDDs of the 7 species investigated in our analysis. These results are basically consistent with the previous studies about PTM in disordered region in protein level^6,31^. The significant higher proportion of PTM sites in IDDs than structured domains found in our analysis indicates that PTM may contribute to the complex functions of IDDs.

### The relationship between DSDR and CDRN

**Figure 5:**
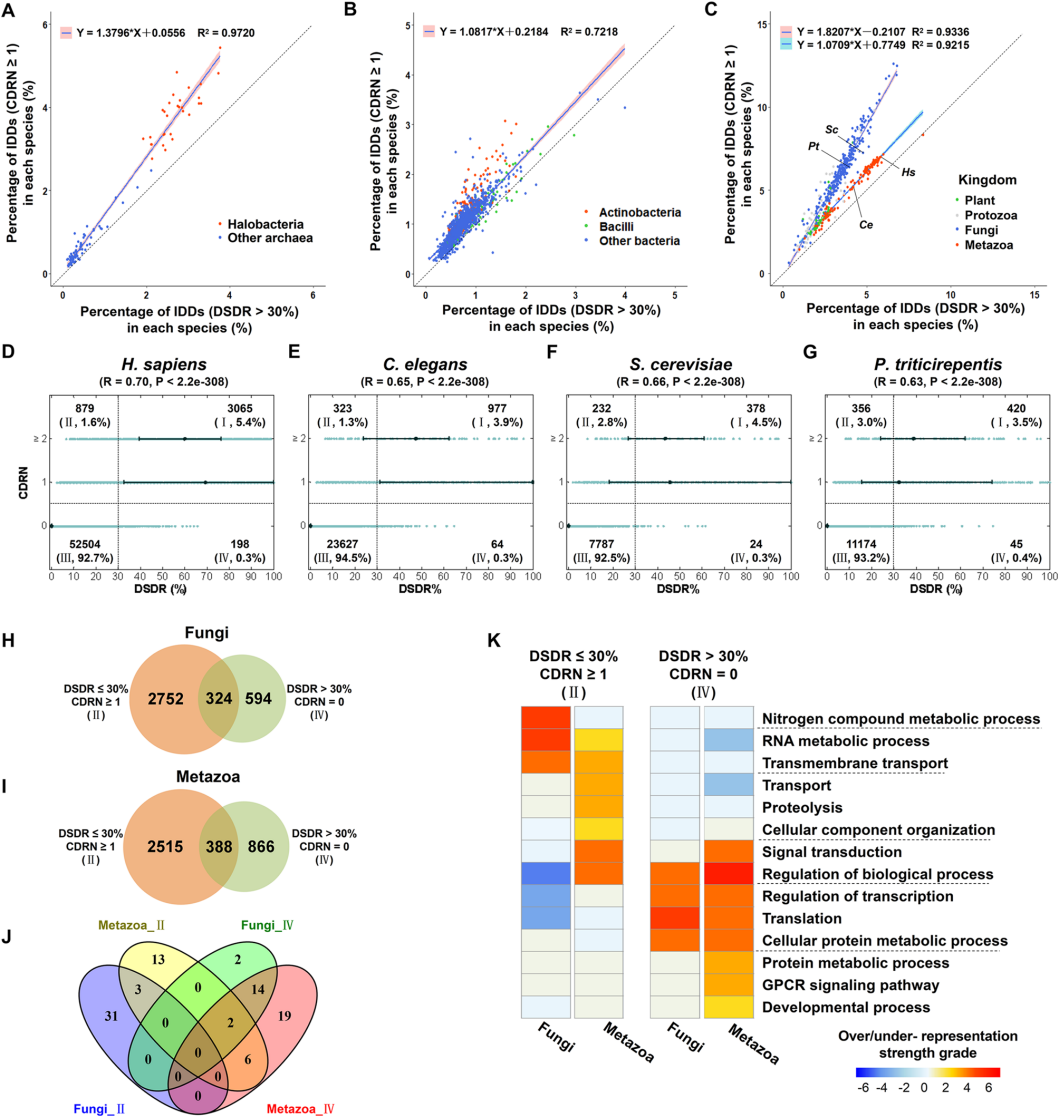
The relationship between DSDR and CDRN and the functions of the special categories. (**A**–**C**) Scatter plots of the percentage of IDDs in each species of the three superkingdoms (**A**, archaea; **B**, bacteria; **C**, eukaryotes). Each point represents a species. The dashed diagonal line represents the line where Y and X are equal. The equation in each sub-figure was obtained by fitting a straight line based on the data of all species of archaea (**A**), bacteria (**B**) and fungi or metazoa (**C**). (**D**–**G**) Scatter plots of the DSDR and CDRN values of the four example eukaryote species. Each point represents a domain. According to the values of DSDR and CDRN, all of protein domains were divided into 4 classes: I, II, III, IV. The sum of the percentages of class I and class IV domains is equivalent to the X value of the corresponding species in sub-figure C; the sum of the percentages of class I and class II domains is equivalent to the Y value of the corresponding species in sub-figure C. (**H**–**I**) Venn diagrams of the IDDs of the two classes (II, IV) of fungi (H) and metazoa (I). There is an overlap between the two classes because some of IDDs belong to different classes (II, or IV) in different species. (**J**) Venn diagrams of the significantly over-represented BP terms for the two classes (II, IV) of IDDs of fungi and metazoan. (**K**) Over- or under-representation analysis of BPs for the two classes (II, IV) of IDDs of fungi and metazoa. The methods of over- or under- representation analysis and the criterion for strength grading are the same as that of Fig. 3A. The dashed lines separate the significantly over-represented BP terms for each class.

In this study, two related, but different measures of the degree of domain disorder were used: (1) the proportions of each grade of domain structural disorder ratio (DSDR); (2) the number of consecutive disordered regions (CDRN). The relationship between DSDR and CDRN was explored in the three superkingdoms. We calculated the proportion of IDDs in each species according to DSDR or CDRN. Scatter plots of the percentage of IDDs in each species show a very interesting phenomenon: The data points of both archaea (Fig. 5A) and bacteria (Fig. 5B) can be fitted into a straight line, while the data points of different kingdoms in eukaryotes, *e.g.* fungi and metazoan, clearly form two lines (Fig. 5C). Similar results were obtained at the domain family level (Figure S15). But there are no such obvious two distinct lines in the scatter plots of Eukaryota in non-domain regions (including linkers, and N- or C- terminal regions) (Figure S16). This reveals that the interesting difference between fungi and metazoans in the relationship between SDR and CDRN exists specifically in domain regions. In other words, the difference in protein disorder pattern between fungi and metazoans are in domains, not in non-domain regions.

According to the values of DSDR and CDRN, all of protein domains were divided into 4 classes: I, II, III, IV (Fig. 5D–G). The slope of the straight line formed by the data points of fungi is greater than that of metazoan (Fig. 5C). Consistent with this result, the differences between the percentage of class II domain (DSDR ≤ 30%, CDRN ≥ 1) and that of category IV (DSDR > 30%, CDRN = 0) of each species in metazoan (Fig. 5D,E) are much lower than those differences in fungi (Fig. 5F,G). The numbers of domains belonging to these two categories in fungi (Fig. 5H) and metazoan (Fig. 5I) were calculated and their biological functions were explored by the over-representation analysis of the biological process (BP). It’s obvious that there is almost no overlap between the significant over-represented BP terms of class II and IV domains (Fig. 5J). Comparison between fungi and metazoans reveals their common and specific significant over-represented BP terms (Fig. 5K), which indicates that some specific metabolic processes in fungi require the class II domains. For example, two fungi-specific class II domains, fungal_trans and PDEase_II, are involved in the regulation of transcription and nitrogen compound metabolic process. Although these two domains are not high-DSDR domains, they both have a consecutive disordered region (29 and 58 amino acid residues, respectively), which is of great significance for their fungi-specific functions (Figure S17, see supplementary descriptions for details). These results suggested that some IDDs with a special relationship between DSDR and CDRN are required in specific clades to perform clade-specific functions. For example, some special functions in fungi require disordered domains, especially those with CDR but not high DSDR values.

### The variation of disorder degree of repeating domains and its functional significance

**Figure 6:**
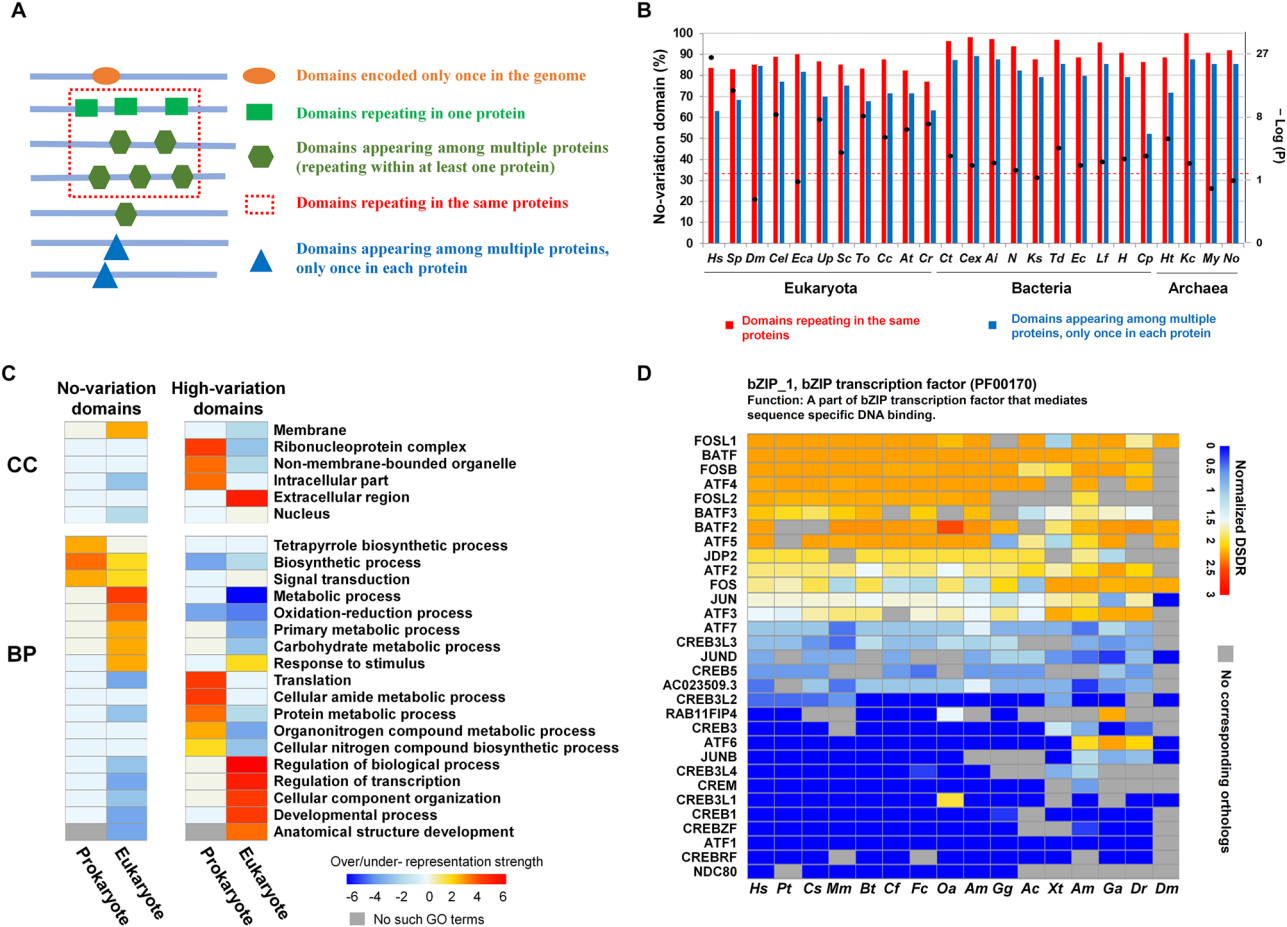
The distribution and functional features of domains with different DSDR variations. (**A**) Classification of protein domains according to the domain distribution in all of the proteins encoded by the genome. (**B**) Distribution of No-variation dominant categories of DSDR variation in the domains repeating in the same proteins and the domains appearing among multiple proteins of the 25 representative species. The DSDR variation value was calculated by the difference of normalized DSDR. Black spots represent statically significant differences, tested by the Fisher’s exact test. Black spots above the dashed line represent significant over representations. The abbreviations: *Hs Homo sapiens; Sp Strongylocentrotus purpuratus; Dm Drosophila melanogaster; Cel Caenorhabditis elegans; To Thalassiosira oceanica; Cc Cyclospora cayetanensis; Eca Enterospora canceri; Up Umbilicaria pustulata; Sc Saccharomyces cerevisiae; At Arabidopsis thaliana; Cr Chlamydomonas reinhardtii; Ct Chloroherpeton thalassium; Cex Caldisericum exile; Ai Alistipes inops; N Nitrospina sp. SCGC_AAA799_A02; Ks Kytococcus sedentarius; Td Thermosulfurimonas dismutans; Ec Escherichia coli; Lf Leptospirillum ferriphilum; H Hydrogenivirga sp. 128–5-R1-1; Cp Chlamydia psittaci; Ht Haloterrigena turkmenica; Kc Candidatus korarchaeum cryptofilum; My Metallosphaera yellowstonensis; No Candidatus nitrocosmicus oleophilus.* (**C**) Over- or under-representation analysis of biological process (BP) and cellular component (CC) for the ‘no-variation’ (DSDR variation = 0) and ‘high-variation’ (DSDR variation > 1) domains (see Materials and methods for details). The methods of over- or under- representation analysis and the criterion for strength grading are the same as that of Fig. 3A. (**D**) Examples of ‘high-variation’ domains. DSDR was normalized by interpolation method. Heatmap shows the normalized DSDR values of the same domain family in different proteins of different species. Each column represents a different species, and each row represents the corresponding orthologs. The abbreviations of the species: *Hs Homo sapiens; Pt Pan troglodytes; CS Chlorocebus_sabaeus; Mm Mus musculus; Bt Bos Taurus; Cf Canis familiaris; Fc Felis catus; Oa Ovis aries; Am Ailuropoda melanoleuca; Gg Gallus gallus; Ac Anolis carolinensis; Xt Xenopus tropicalis; Am Astyanax mexicanus; Ga Gasterosteus aculeatus; Dr Danio rerio; Dm Drosophila melanogaster*.

According to the protein domain repetition features, all the protein domains were divided into four categories (Fig. 6A). A comparison of different domain categories within the same species revealed that the percentages of IDDs were higher in the domains encoded only once in the genome than those repeating in the same or different proteins (Figure S18A,B, Table S6). This result indicates that gene-specific non-repeating protein domains prefer to be IDDs compared to repeating domains, especially in eukaryotes. This type of relationship between domain repeating pattern and domain disorder degree may be the result of their relationship with domain age. As the result in Fig. 3 B,C, young domains, especially eukaryote-specific domains, tend to be disordered; young domains also tend to have fewer repetitions (Figure S18C) due to the smaller number of events of duplication and shuffling since their emergence during evolution. Thus, special functional requirements (Fig. 4B) led to the emergence of new IDDs, which have fewer repetitions.

According to the common understanding, the same domain family may share the same or a similar structure; thus, their degree of disorder may be similar. Surprisingly, the DSDR values of some repeating domains from the same domain family are completely different. Thus, we investigated the variation of disorder degree of all repeating domains in each whole proteome to get a complete understanding of this phenomenon and its evolutionary and functional significance. The DSDR values were normalized using the interpolation method (Figure S19A, see Materials and Methods). The variation values of DSDR were divided into the following three classes: 0 (no-variation), 0–1 (low-variation), and 1–3 (high-variation). The repeating domains were divided, using the dominant category method, into the following four classes: ‘no-variation’, ‘low-variation’, ‘high-variation’ and ‘no dominant category’ (Figure S19B). The proportions of each dominant category were calculated for the 25 species (Table S7). It was found that the majority of repeating domains belonged to ‘no-variation’ and ‘low-variation’ categories. We next explored the direct contributing factors for the variation of the disorder degree and the causes for the low proportion of ‘high-variation’ category. The results revealed that the DSDR value of a domain is mainly determined by the hydrophobicity of the amino acid residues within or close to the domain regions (Figure S20, Table S8, see supplementary descriptions for details).

Amino acids have different hydrophobicity; therefore, if the sequences of two protein domains are similar, it would be expected that they have similar hydrophobicity and therefore, similar DSDR values; however, sometimes, different amino acids also have similar hydrophobicity. Therefore, to fully understand the reasons for DSDR variation, the relationship between sequence similarity (Dataset 4) and DSDR variation degree was explored. We found that a large proportion of repeating domain pairs have a sequence similarity of less than 40% (Figure S21A–D), which verifies the conclusion of the low level of sequence similarities between domain repeats^44^. Repeating domains that had a low sequence similarity exhibited the low variable DSDR values (< 1) (Figure S21A–D), that is, such domains had similar DSDR values. These results indicate that sequences of repeating domains are not conserved, but their structures are conserved. Low sequence similarity can prevent the aggregation of the same domains, and thereby prevent protein misfolding^45^. In contrast, a low variation of DSDR ensures that proteins take on the correct conformation and that their functions are conserved.

Although only a small part of the repeating domains has high variations of DSDR values, it’s of great significance to explore the distribution pattern of the variation of DSDR across different species and to decipher the functional difference between the domains with constant and high-variable DSDR values. The number of ‘high-variation’ domains in a species has a significant positive correlation with organism complexity. But after normalized by the total number of repeating domains, this correlation disappeared (Table S9, supplementary results). We next compare the proportion of ‘no-variation’ dominant category among 25 representative species (Fig. 6B, S21E). The domain families repeating in the same proteins had a higher proportion of the ‘no-variation’ dominant category than the domain families repeating amongst multiple proteins in each species (Fig. 6B, S21E). Furthermore, the differences in the proportion of the ‘no-variation’ dominant category in different repetition categories were confirmed by the Fisher’s exact test. Twenty of the twenty-five species showed significant differences between the two types of repeating domains (Fig. 6B, S21E).

The DSDR variation of the protein domains is closely related to their functions. We found the functions of ‘no-variation’ and ‘high-variation’ domains are quite different which may reveal the functional constraints on DSDR variations (Fig. 6C, Table S10). ‘No-variation’ domains of prokaryotes tend to localize at membrane and function in tetrapyrrole biogynthetic process and signal transduction. In eukaryotes, ‘No-variation’ domains tend to localize at membrane and function in metabolism process, oxidoreduction, response to stimulus. For example, PsaA_PsaB domain (pfam00223), participating in photosynthesis, a branch of metabolic process, shows a constant degree of disorder (DSDR = 0) in different plants (Figure S21F). PsaA and PsaB are specifically located at the chloroplast thylakoid membrane^46^. The stable three-dimensional structure of the PsaA_PsaB domain ensures that PsaA and PsaB can specifically recognize and bind to P700 to form a trimeric complex (photosystems I, PSI)^47,48^, acting as a plastocyanin/cytochrome c6-ferredoxin oxidoreductase in the photosynthesis.

High-variation domains in prokaryotes tend to localize at intracellular part and function in translation and protein metabolism. In contrast, high-variation domains in eukaryotes tend to localize at extracellular region and nucleus participating in many regulation processes, such as transcription regulation. This type of domains needs variable DSDR values to meet with the diversified functions. For example, bZIP_1, a bZIP transcription factor, has a wide variety of DSDR values (from 0 to 82.5% in human) (Fig. 6D). Its varied disorder degree contributes to the functional versatility of transcription factors. The flexibility of bZIP_1 domain of AP-1 family enables them to freely combine to form different heterodimers, and then combine to synthesize different DNA locations to realize functional diversification. While the disorder of the ZIP domain in the CREB family remains stable. This helps them bind to specific regions of DNA precisely.

## Discussion

IDPs play important roles in various biological processes^1–3^ and they usually have special evolutionary features^1,2^. For example, genes encoding IDPs tend to be evolutionarily young^49^; the disordered regions in IDPs tend to have higher amino acid replacement rates^50^ and biased amino acid composition affected by the GC content^51^. In general, these disordered regions in proteins are usually the linking regions between different protein domains^20,51^. However, IDDs indeed exist. The functions of IDDs have been investigated in previous studies^12,52–55^, but the comprehensive analysis of the distribution and functional significance of IDDs is lacking. In this study, we investigated the functional and evolutionary characteristics of IDDs at the entire proteome level across three superkingdoms. Our analyses revealed that the degree of domain disorder is not only related to organism complexity, but is also related to the clade-specific special functions.

Some previous studies have investigated the contribution of protein disorder to the formation of complex regulation mechanisms in eukaryotes. For example, evolution from unicellular organisms to multicellular organisms in all major eukaryotic lineages significantly benefited from IDPs, alternative splicing (AS) and post-translational modification (PTM), in which IDPs conferred multifunctionalities and tightly associated with AS and PTMs^55,56^. Nido *et al.* found that protein disorder degree in the centrosome correlates with species complexity^57^. Yruela *et al.* explored the role of disordered proteins in chloroplast and nucleus of plants^58^.

From the general viewpoint, it was reported that there is a significant positive correlation between the disorder content of proteins and organism complexity measured by cell type numbers^55^. However, in that analysis^55^, the number of intrinsically disordered protein residues was not normalized by the total size of proteome. The more complex the species, the larger the total amount of amino acid residues, and the more disordered residues^26^. Therefore, it can’t be said that the increase of disordered residues contributes to the species complexity. If we want to evaluate the contribution of protein disorder to the complexity of organisms, we should use the percentage of disordered residues to total residues for correlation analysis. By this way, both Schad’s^26^, Xue’s^27^ and our results indicated that there is no significant correlation between biological complexity and protein disorder in eukaryotes. In Yruela *et al*.’s work, they also reported that if they use the fraction of disordered residues in transcription factor sequences to analyze the correlation with organism complexity, the correlation was poor^59^. For the first time, our analysis revealed that the degree of disorder of protein domains correlates with organismal complexity with a significant correlation coefficient. The reason for this phenomenon may lie in the more important functionality of protein domains than other regions of proteins. The protein domains are the structural and functional units of proteins. If a protein domain has a disordered region or has a high proportion of disordered residues, the structure and function of this domain will be more flexible, which in turn has a strong influence on the function of proteins. Thus, complex organisms need more intrinsic disorder domains to perform complex regulatory functions.

However, not all species are in line with the general positive correlation between the degree of domain disorder and organism complexity. For example, halobacteria, ascomycetes and green algae have higher proportions of IDDs than the species with similar or even higher organism complexity. These phenomena indicate that the degree of disorder of protein domains is also influenced by certain special functions evolved in a specific evolutionary lineage. In halobacteria, the protein adaptations toward the high-salt pressure result in special characteristics of the protein sequence : the acidic and polar amino acid residues are over-represented significantly, and the hydrophobic residues are remarkably under-represented^60^. Studies have shown that halophiles prefer coil formation to alpha-helix formation^61^. Halophiles may rely on disordered domains to maintain their adaptability to high-salt environments. In eukaryotes, the virulence of ascomycetes and the cilium formation of green algae is also regulated by the functions of a large number of disordered domains.

As for the causes of formation of disordered protein domains, the long length of the domain maybe a prerequisite for it to have a CDR. We found that the IDDs having a CDR but a low disorder ratio (DSDR < 30%) (Class II) tend to be much longer than those containing higher disorder ratio (DSDR > 30%) but no CDR (Class IV) (Figure S17A and supplemental description in additional file 1). Further, we compared all the domains with various disorder degrees (Figure S22). In eukaryotes, the domains containing CDR(s) tend to be longer than those without a CDR (Figure S22C). This is consistent with the above result (Figure S17A). Interestingly, from the viewpoint of DSDR, the domains containing a small fraction of disorder residues (0 < DSDR ≤ 10%) tend to be the longest type (figure S22D–F). It indicates that long protein domains tend to have at least a CDR but not to have a large fraction of disorder residues. But this relationship is not the cause-effect relationship. Both of the length and disorder degree of protein domains are the results of evolution. The functional requirements played driving roles during the evolutionary processes that the longer and disordered domains were remained to perform complex functions.

In the viewpoint of the disorder variation of the same protein domains, the clade-specific ‘no-variation’ and ‘high-variation’ domains may contribute to the clade-specific functions which need constant or variable DSDR values respectively.

Notably, although the accuracy of the disorder prediction methods has been greatly improved these years, it is still difficult to guarantee 100% accuracy. This may not affect the core conclusion of large-scale statistical analysis, as in this study. In addition, we used two disorder prediction methods based on different principles and similar conclusions were obtained. Thus, the core conclusions of this study are reliable. However, if we want to explore the functions of a certain IDD in a certain protein, we should do experiments to confirm its structural properties. Another limitation in this analysis is the number of species having cell type number data. But the 51 species are also representative across eukaryotes, including deuterostomes (16 species), protostomes (4 species), sponges and cnidarians (3 species), protists (6 species), fungi (8 species), plant (13 species).

In conclusion, this study focused on the uneven distribution pattern and functional characteristics of the IDDs across 1870 species of the three superkingdoms. We found that intrinsic disorder of protein domain contributes to both of organism complexity and clade-specific functions: 1) complex species tend to have higher proportions of IDDs, contributing to the increasing organism complexity; 2) some clade-specific IDDs, clade-specific domains with specific DSDR/CDRN ratios and clade-specific ‘no-variation’ or ‘high-variation’ domains are involved in clade-specific functions. These new findings enrich our knowledge about the evolution of protein domains and provide valuable new insights for the functional studies of disordered domains.

## Methods

### Datasets

The protein sequences were retrieved from the Ensembl Database^62^ (V90, ftp://ftp.ensembl.org/pub/release-90/fasta/), Ensembl Metazoa Database (V43, ftp://ftp.ensemblgenomes.org/pub/release-43/metazoa/fasta), Ensembl Bacteria Database (V39, ftp://ftp.ensemblgenomes.org/pub/release-39/bacteria//fasta/), Ensembl Plants Database (V42, ftp://ftp.ensemblgenomes.org/pub/release-42/plants/fasta/), Ensembl Fungi Database (V44, ftp://ftp.ensemblgenomes.org/pub/fungi/release-44/fasta/), Ensembl Protists Database (V44, ftp://ftp.ensemblgenomes.org/pub/protists/release-44/fasta/), Ensembl Tardigrades Database (http://download.tardigrades.org/v1/sequence/) and NCBI Refseq Database (ncbi.nlm.nih.gov/protein)^63^.

### Identification of protein domains and the definition of domain family

The protein domains were identified as described previously^36,40^. In brief, to identify the domains presented in the whole sequences of each species, we used HMMER-scan program^64^ to search the Pfam-A databases^65^. And the full sequence E value and C-Evlaue were kept less than 0.01. According to previous definitions^32,33^, domain families in our study refer to the unique domain types. Protein domains belonging to a same family share the same Pfam IDs and domain names, but are located in different contexts, including different proteins and different regions of the same protein.

### Intrinsic disorder prediction

Intrinsically disordered regions were identified using SPOT-D^66^(default parameters) and IUPred^67^ (V1.0, Prediction type: long disorder). SPOT-D and IUPred are popular programs for the prediction of structural disorder scores. SPOTD is based on deep recurrent and convolutional neural networks. IUPred is based on the physicochemical properties of amino acids. Both of them were used to calculate the structural disorder value of each amino acid. An amino acid with a high structural disorder value which exceeds the default threshold was considered to be disordered. ESpritz^68^ (V1.1, PSI-BLAST based prediction, Prediction type: NMR, Decision threshold: 5% False Positive Rate) was used as a corrected method for halobacteria. PSI-BLAST can avoid over-prediction of disorder scores^35^.

To further validate the main conclusions, a consensus predictor, MobiDB-lite (http://mobidb.bio.unipd.it/)^69^, was used. The protein sequence and disorder region information were downloaded using API method (https://mobidb.bio.unipd.it/help/apidoc). 39 species were included in the analysis of the correlation between disorder degree and organism complexity (Table S1C). 25 representative species were used to validate the main conclusions about the disorder distribution and variation (Table S1C, S2C).

### Calculation and classification of DSDR and CDRN

According to the results of domain identification and intrinsic disorder prediction, we extracted the corresponding disorder value of each amino acid of each domain. The DSDR was represented by the percentage of the disordered amino acids in a protein domain. In a previous study^70^, proteins were divided into three categories according to PSDR: 0–10%, ‘highly structured’; 10–30%, ‘moderately unstructured’; 30–100%, ‘highly unstructured’. Similarly, the protein domains were divided into four classes according to the DSDR values: 0, ‘completely structured domain’; 0–10%, ‘highly structured domain’; 10–30%, ‘moderately unstructured domain’; and 30–100%, ‘intrinsically disordered domain’.

The definition of CDRN is more complicated. For domains with more than 50 amino acids, one region with more than 20 consecutive disordered residues is a consecutive disordered region (CDR). For the rest of the domains, if the number of consecutive disordered residues in a region is more than 40% of the total number of the domain then this region is considered as a consecutive disordered region. From the viewpoint of CDRN, the protein domains were divided into three classes: 0, ‘domain without CDR’; 1, ‘domain with one CDR’; ≥ 2, ’domain with multiple CDRs’. High-DSDR (DSDR > 30%) domains and domains containing one or more CDRs are considered as IDDs. Notably, this definition of IDD is different from the ‘disordered’ type in Pfam database. In Pfam, only fifty-five entries with more than 100 residues and conserved sequences in primate proteins are regarded as ‘disordered’^71^. But there are also disordered regions in other types, such as PF03250 in ‘family’ type^72^. IDDs in this study generally refer to the domains with high DSDR or containing CDR in all Pfam entries types. Disordered width, in this study, means the percentage of species in which the domain is an IDD among the species containing this type of domain.

### Normalization of DSDR by interpolation method

The DSDR values were normalized using the interpolation method. Four points, (0, 0), (10, 1), (30, 2) and (100, 3) were used to calculate the coefficients *a*, *b* and *c*, and the constant *d*, of the equation $y = a \times x^{3} + b \times x^{2} + c \times x + d$. The four pair values of these four points were used to replace the variables *x* and *y* in the equation respectively, and then, the following values were determined: *a* = 1/78,750, *b* =  − 137/63,000, *c* = 253/2100 and *d* = 0. Therefore, the normalization equation can be expressed as follows:

$$y = \frac{{x^{3} }}{78750} - \frac{{137x^{2} }}{63000} + \frac{253x}{{2100}}$$

where *x* represents the DSDR value, and y denotes the normalized DSDR value.

The normalized curve is shown in Figure S19A.

### Dominant category method

Each domain family was assigned a DSDR dominant category according to the DSDR distribution of the domain family. As mentioned above, DSDRs were divided into four grades. For each domain family, the number and proportion of each DSDR grade was calculated. If the proportion of a DSDR grade was greater than 0.5 then the domain family was assigned to this DSDR dominant category (Figure S2). According to the DSDR variation values calculated using the domain pairs (the difference value of the normalized DSDRs) (Figure S16B), we divided DSDR variation values into three levels: 0 (no-variation domain), 0–1 (low-variation domain) and 1–3 (high-variation domain). DSDR variation dominant categories were defined in a similar way (Figure S16C).

### Definition of organism complexity

Organism complexity was measured by the cell type number in a species^26,37^ (Table S9B). We collected the cell type number data of 51 species.

### Domain age

We defined the domain age by the oldest taxon in which the protein domain first appeared in the Pfam species tree. To get the species in which one protein domain first appeared, a previous procedure was performed^40,73^. Domains of 25 representative species are divided into different age grades.

### PTM data retrieving and calculating

PTM data were retrieved from the Swiss-Prot database (release 2020_06, https://www.uniprot.org/downloads). Due to the limitation of the current known PTM information in the database, only a few representative model species could be included in this analysis. The text format file of Swiss-Prot was downloaded and the data of seven species (*Homo sapiens*, *Pongo abelii*, *Mus musculus*, *Bos taurus*, *Drosophila melanogaster*, *Saccharomyces cerevisiae*, *Arabidopsis thaliana*) were extracted from it. The MOD_RES type information was selected from FT (Feature Table) lines. Meantime, the protein sequences of these seven species were also extracted from the text format file of Swiss-Prot. The disorder scores were calculated and protein domains were identified using SPOTD and HMMER-scan (mentioned above) respectively. The PTM site number and type in each type of protein domains/regions were calculated for the further comparison and over-representation analysis.

### Calculation of sequence similarity of protein domains

The BLASTp program was used to compare the sequences of the repeating protein domains. Further, Blocks Substitution Matrix 62 (BLOSUM62) was used. The E value threshold was set at the default value of 10. The formula to calculate the sequence similarity degree can be expressed as follows:

$$Sequence\_Similarity = Identity\% \times \frac{{\left[ {100 - ABS(100 - Match\_query\_percent)} \right] + \left[ {100 - ABS(100 - Match\_subject\_percent)} \right]}}{2 \times (100 - Query\_first\_percent)}$$

‘Match_query_percent’ denotes the percentage of the matched sequence in the query sequence. ‘Match_subject_percent’ represents the percentage of the matched sequence in the subject sequence. ‘Query_first_percent’ indicates the percentage of the region from the domain start site to the matched first site in the domain sequence. ‘ABS’ refers to the calculation of absolute values.

### General approach for over- or under-representation analysis

The over- or under-representation analysis of domain GO annotations (Dataset 5) was based on the hypergeometric distribution model and the Benjamini–Hochberg method was used to correct the *P* value^74^. The logarithm of the *P* value (log *P*), positive or negative, was used to represent the strength of the over- or under-representation of proteins containing certain domain characters. When the heat maps were used to represent the over- or under-representation strengths, the values of ± log(*P*) were transformed into 14 grades (− 7 to + 7): − 7, log(*P*) ≤  − 12; − 6, − 12 < log(*P*) ≤  − 9; − 5, − 9 < log(*P*) ≤  − 6; − 4, − 6 < log(*P*) ≤  − 3; − 3, − 3 < log(*P*) ≤  − 2; − 2, − 2 < log(*P*) ≤  − 1.301; − 0.25, − 1.301 < log(*P*) ≤ 0; 0.25, 0 <  − log(*P*) < 1.301; 2, 1.301 ≤  − log(*P*) < 2; 3, 2 ≤  − log(*P*) < 3; 4, 3 ≤  − log(*P*) < 6; 5, 6 ≤  − log(*P*) < 9; 6, 9 ≤  − log(*P*) < 12; 7, − log(*P*) ≥ 12. Note: the grading criterion for figure S8 as not the same as this (see the corresponding figure legend).

## Supplementary Information

Supplementary Information

Supplementary Information2

## Data Availability

All data generated or analyzed in this study are included in this published article and the supplementary information files. Supplementary description of part of results, methods, table S1–S10, and figure S1–S22 are available at *Scientific Reports* online. The raw data in the analyses (Datasets 1–6) are available at http://dis-domain-data.ncpsb.org/.
