# Supplementary material for: Intrinsic disorder in protein domains contributes to both organism complexity and clade-specific functions
Source: Sci Rep. 2021 Feb 4;11:2985. doi: 10.1038/s41598-021-82656-9 (PMC7862400; doi:10.1038/s41598-021-82656-9)
Supplement: Supplementary file 1 — Supplementary Information [file 41598_2021_82656_MOESM1_ESM.pdf]

Intrinsic disorder in protein domains contributes to both organism complexity and clade-specific functions

(Additional information)

Chao Gao[1, #], Chong Ma[1, 2, #], Huqiang Wang[1], Haolin Zhong[1], Jiayin Zang[1], Rugang Zhong[2], Fuchu He[1, *], Dong Yang[1, #, *]

[1] State Key Laboratory of Proteomics, Beijing Proteome Research Center, National Center for Protein Sciences (Beijing), Beijing Institute of Lifeomics, Beijing 102206, China

[2] Beijing Key Laboratory of Environmental and Viral Oncology, College of Life Science and Bioengineering, Beijing University of Technology, Beijing 100124, China

[#] Equal contribution.

[*] Corresponding authors.

Fuchu He, National Center for Protein Sciences Beijing, 38 Science Park Road, Changping District, Beijing 102206, China.

Tel: 8610- 61771001, E-mail: hefc@nic.bmi.ac.cn.

Dong Yang, National Center for Protein Sciences Beijing, 38 Science Park Road, Changping District, Beijing 102206, China.

Tel: 8610-61777051, Fax: 8610-61777050, E-mail: yangdongbprc@163.com.

Contents

Supplemental results

- The special features of the intrinsic disordered domains (IDDs) with a consecutive disorder region (CDR) but a low disorder ratio (DSDR < 30%)
- Which physico−chemical characteristic of amino acid was correlated with values of P matrix?
- The relationship between DSDR variation and organism complexity

Supplemental references

Supplemental figures

Figure S1. Distribution of IDDs (CDRN ≥ 1) across the three superkingdoms (related to figure 1).

Figure S2. Distribution of IDDs (DSDR>30%, or CDRN ≥ 1) across 25 representative species using MobiDB-lite data.

Figure S3. Diagram of ‘dominant category’ approach for the disorder degree classification of domain families.

Figure S4. Calculation of the degree of domain disorder in archaea using different methods, including a corrected method.

Figure S5. Scatter-plot of the median repetition number of completely structured domains (blue triangle) and IDDs (red circle).

Figure S6. The correlation between structural disorder and organism complexity analyzed based on MobiDB-lite data.

Figure S7. Box-plot of the percentage of intrinsically disordered (CDRN ⩾ 1) domains (left panel) or domain families (right panel) in each species across the different phyla of eukaryotes.

Figure S8. The distribution of IDDs in each age grade of the 25 representative species (related to figure 3C).

Figure S9. Figure S9. Comparison of the normalized DSDR values of the same domain families in evolutionarily more complex species and simpler species (related to figure 3D).

Figure S10. The number and functions of special IDDs in archaeal halobacteria.

Figure S11. The special functions of IDDs in Ascomycota and Basidiomycota revealed by the enriched mutant phenotypes.

Figure S12. Over- or under-representation analysis of BPs for IDDs with different ages and different distribution width.

Figure S13. The examples for the domains classified by domain age and disorder width (related to figure S12).

Figure S14. Comparison of the PTM site proportion in different type of domains/regions classified according to disorder degree.

Figure S15. Scatter plot of the percentage of disordered domain families in each species of the three superkingdoms (related to figure 5A-C).

Figure S16. Scatter plot of the percentage of non-domain disordered regions in each

species of the three superkingdoms (related to figure 5A-C).

Figure S17. The special features of the IDDs with one or more consecutive disorder region (CDR) but a low disorder ratio (DSDR<30%) (related to figure 5K).

Figure S18. The distribution of IDDs and young domains in different repeating domain categories.

Figure S19. The method for the normalization of DSDR values and the classification of DSDR variation.

Figure S20. Correlation between the values of physico−chemical characteristics of amino acid and the values in P matrix.

Figure S21. The correlation between DSDR variation values and domain sequence similarity and supplemental results about the distribution and functional features of domains with different DSDR variations.

Figure S22. Comparison of domain length among the protein domains with different disorder degrees.

Supplemental tables

Note: The tables S1-S10 are in an additional Excel file.

Table S1. Domain numbers and their percentages in different disorder grades across the three superkingdoms. DSDRs were divided into two grades according to values (≤ 30% and > 30%). CDRNs were also divided into two grades (0, ≥ 1). Domain numbers and percentages of each disorder grade were calculated in each species. SPOT-D (A), IUPred (B) were used for disorder prediction of 1870 species, and the disorder

data of 58 species from MobiDB-lite (C) were also used for validation analysis.

Table S2. Domain family numbers and their percentages in different disorder grades across the three superkingdoms. Each domain family was assigned with a dominant category based on dominant category approach (see Materials and Methods). The numbers and the percentages of domain families of different disorder dominant categories were calculated in each species. SPOT-D (A), IUPred (B) were used for disorder prediction of 1870 species, and the disorder data of 58 species from MobiDB-lite (C) were also used for validation analysis.

Table S3. Colour description of each phylum and class in figure 1A and supplemental figure 1A.

Table S4. Median domain repetition counts in different disordered grades in each species (related to figure S5). For each species, the median repetition counts of all the domain families at each disorder grade were calculated. SPOT-D (A) and IUPred (B) are used for disorder prediction.

Table S5. The significantly over-represented PTM types in intrinsically disordered domains.

Table S6. Domain numbers and percentages of different disorder grades in different domain categories classified by domain repetitions. SPOT-D (A) and IUPred (B) are used for disorder prediction.

Table S7. Summary of DSDR variation in twenty-five representative species (related to figure 6B). SPOT-D (A) and IUPred (B) are used for disorder prediction.

Table S8. The raw data of correlation analysis between physico – chemical

characteristics and P matrix values.

Table S9. The correlation between DSDR variation and organism complexity. A1, B1. The results of the correlation analysis between DSDR variation and organism complexity. A2, B2. The raw data for the correlation analysis between DSDR variation and organism complexity. SPOT-D (A) and IUPred (B) are used for disorder prediction.

Table S10 Detailed information of the no-vairation and high-variation domains in twenty-five representative species.

Datasets

Dataset 1. The disorder values calculated using SPOT-D and IUPred for each amino acid residue of all proteins in the 1870 species across 3 superkingdoms. The scores of each residue were shown according to the order of each protein sequence. The letters represent the residues in protein sequences.

Dataset 2. DSDR and CDRN values of each domain in the 1870 species.

Dataset 3. The median normalized DSDR values of all protein domain families in each species across three superkingdoms (related to Figure 3A). DSDR was normalized by the interpolation method (see *Methods* section). The normalized DSDR values of the same domain family within one species is represented by the median value. "NA" means no such domain in this species.

Dataset 4. Sequence similarities for the domain repeats of 25 representative species. In the title line of each table, 'PrID_DomID_SiteID-1-PrID_DomID_SiteID-2' denotes each domain pair. 'Degree_of_variation' represents the variation of DSDR.

Dataset 5. Domain GO annotations used in the study.

These five datasets are available at http://dis-domain-data.ncpsb.org/.

Supplemental results

The special features of the IDDs with one or more consecutive disorder region (CDR) but a low disorder ratio (DSDR < 30%)

The comparison of domain disorder type between fungi and metazoans reflected the difference of domain disorder characteristics between them. Fungi have more class II IDDs, which have a consecutive disorder region (CDR) but a low disorder ratio (DSDR<30%). From the viewpoint of domain length, it is clear that class II IDDs are significantly longer than class IV IDDs (Figure S17A). The long peptide chains of the class II IDDs tend to contain a continuous disordered region (CDR), which will increase its flexibility to form more flexible conformations via the disordered linker region. For example, fungal_trans domain (a fungi-specific domain) in the transcriptional activator SEF1 of *Saccharomyces cerevisiae* contains a CDR (40 aa, from Asp^{523} to Glu^{551}), which is located in the middle region of fungal_trans domain (from Thr^{357} to Ile^{577}) (Figure S17B). The structure of SEF1 was modeled using SWISS-MODEL [1-5]. The CDR in the fungal_trans domain is seated between two coils, which is similar to a flexible chain linking two compact rods (Figure S17C). It implies that the CDR in fungal_trans domain may play as an entropic chain or a linker to adjust the local conformation to bind to DNA as SEF1 is a transcription factor [6].

Another fungi-specific class II domain, cAMP phosphodiesterases class-II (PDEase_II) domain in Nadsonia fulvescens with a long CDR (45 aa, Figure S17D, E), is involved in heterocyclic metabolic process. PDEase_II catalyzes the hydrolysis of

cAMP to 5' nucleoside monophosphate. CDR may regulate this process by regulating the flexibility of the domain. Interestingly, the segment from amino acid position 209 to 258 of this protein, which highly coincides with the CDR (203-260), is predicted to be a polar region by MobiDB-lite [7]. Since this protein can catalyse the hydrolysis of cAMP, the disordered polar region may be responsible for increasing the affinity of PDEase_II to water molecules.

Which physico−chemical characteristic of amino acid was correlated with values of P matrix?

The degree of disorder is determined by the physical-chemical properties of amino acid residues within the domain regions and the amino acid residues close to the domain regions [8]. The relationship between the amino acid physical-chemical properties and the values of the P matrix used in the IUPred [8, 9] method was investigated (Table S8). The correlation between values in P matrix [8] and the average values of the hydrophobicity index (Table S8) was significantly negative ($R = -0.47$, $P = 6.8e-013$, Figure S20A). But there are no such significant correlations between values in P matrix and average values of molecular weight (Figure S20B) or isoelectric points (Figure S20C). This result indicated that hydrophobicity of amino acid residues plays important roles in determining the degree of disorder.

The relationship between DSDR variation and organism complexity

Fifty-one eukaryotes were selected for correlation analysis. We found that there was a significant positive correlation between the number of ‘high-variation’ domains and the

number of cell types, which indicates that variation of the disorder degree of protein domains substantially contribute to organism complexity (Table S9). We speculated the high variation of domain disorder may lead to its versatility and regulate the complex signal networks more precisely. However, after normalized by the total number of repeating domains, this correlation disappeared. We can see from table S9 that there is a stronger correlation between the number of repeating domains and organism complexity. Thus, if we calculate the correlation between the percentages of 'high-variation' domains and cell type numbers in a species, there is no significant correlation.

Supplemental References

1. Guex, N., M.C. Peitsch, and T. Schwede, *Automated comparative protein structure modeling with SWISS-MODEL and Swiss-PdbViewer: a historical perspective.* Electrophoresis, 2009. **30 Suppl 1**: p. S162-73.
2. Benkert, P., M. Biasini, and T. Schwede, *Toward the estimation of the absolute quality of individual protein structure models.* Bioinformatics, 2011. **27**(3): p. 343-50.
3. Bienert, S., et al., *The SWISS-MODEL Repository-new features and functionality.* Nucleic Acids Res, 2017. **45**(D1): p. D313-D319.
4. Waterhouse, A., et al., *SWISS-MODEL: homology modelling of protein structures and complexes.* Nucleic Acids Res, 2018. **46**(W1): p. W296-W303.
5. Bertoni, M., et al., *Modeling protein quaternary structure of homo- and hetero-oligomers beyond binary interactions by homology.* Sci Rep, 2017. **7**(1): p. 10480.
6. van Peij, N.N., J. Visser, and L.H. de Graaff, *Isolation and analysis of xlnR, encoding a transcriptional activator co-ordinating xylanolytic expression in Aspergillus niger.* Mol Microbiol, 1998. **27**(1): p. 131-42.
7. Necci, M., et al., *MobiDB-lite: fast and highly specific consensus prediction of intrinsic disorder in proteins.* Bioinformatics, 2017. **33**(9): p. 1402-1404.
8. Dosztanyi, Z., et al., *The pairwise energy content estimated from amino acid composition discriminates between folded and intrinsically unstructured proteins.* J Mol Biol, 2005. **347**(4): p. 827-39.
9. Habchi, J., et al., *Introducing protein intrinsic disorder.* Chem Rev, 2014. **114**(13): p. 6561-88.

Supplemental figure legends

Figure S1. Distribution of IDDs (CDRN ≥ 1) across the three superkingdoms (related to figure 1). A. The percentage of IDDs (CDRN ⩾ 1) in each species from archaea (light green), bacteria (lime) and eukaryotes. Each bar indicates the results of a species. All species are arranged according to taxonomy information from NCBI database. The superkingdoms are separated by solid lines and the kingdoms in eukaryotes, including protists (light blue), metazoa (aqua), fungi (yellow) and plant (pale goldenrod), are separated by dashed lines. Each phylum is filled with different colors in the middle circle. The outer circle represents different classes. The detailed information about the names and colors of each species, class and phylum is presented in supplementary table S1-S3. The abbreviations in this figure: Ha, the class of Halobacteria; CDRN, consecutive disordered region number. B. Box-plot of the percentage of intrinsically disordered (CDRN ⩾ 1) domain (purple) or domain families (red) in each species across the three superkingdoms (C, excluding halobacterias). For box-plots, the values of upper and lower quartile are indicated as upper and lower edges of the box, and the median values are indicated as a bar in the box. The differences in the percentage of disordered domain distribution between different categories are examined by Mann–Whitney U test. The corrected *P* values are shown at the top of each panel. D. Cumulative probability of the percentage of intrinsically disordered domain (solid line) or domain family (dashed line) in archaea (blue), bacteria (green) and eukaryotes (red) (E, excluding halobacterias).

Figure S2. Distribution of IDDs across 25 representative species using MobiDB-lite data. Box-plot of the percentage of intrinsically disordered (DSDR>30% in A, B, or CDRN ⩾1 in C, D) domain (purple) or domain families (red) in each species (B, D, excluding halobacterias). For box-plots, the values of upper and lower quartile are indicated as upper and lower edges of the box, and the median values are indicated as a bar in the box. The differences in the percentage of disordered domain distribution between different categories are examined by Mann–Whitney U test. The corrected *P* values are shown at the top of each panel.

Figure S3. Diagram of 'dominant category' approach for the disorder degree classification of domain families. The yellow rectangles represent one domain family and they have eight repeats among three proteins. Each repeat has a DSDR value and a CDRN value. We calculated the percentage of each DSDR and CDRN grade and the dominant grade (> 0.5) was the DSDR and CDRN dominant category of this domain family, which represented the disordered feature of this domain family.

Figure S4. Calculation of the degree of domain disorder in archaea using different methods, including a corrected method. Box-plots show the percentage of disordered domain in each species in different phyla of Archaea. SPOT-D (A and B), IUPred (C and D), ESpritz (E and F) and ESpritz (PSI-BLAST) (G and H) were used for intrinsic disorder prediction. Both results based on DSDR (A, C, E and G) and CDRN (B, D, F and H) are shown.

Figure S5. Scatter-plot of the median repetition number of completely structured domains (blue triangle) and IDDs (red circle). Each species has two corresponding

domain repetition counts. The numbers of domains are shown on the label of the X-axis (number of species in which completely structured domain repetition counts is larger than more disordered domain repetition counts/number of all species in each superkingdom).

Figure S6. The correlation between structural disorder and organism complexity analyzed based on MobiDB-lite data. (A) Protein disorder degree was measured by the average of all representative proteins (the longest one for each gene) in each species. **(B)** Proteins with more than 30% disordered amino acids (PSDR > 30%) were regarded as disordered proteins. Protein disorder degree was measured by the percentage of disordered proteins in each species. **(C, D)** At domain level, structural disorder of each species was measured by the percentage of IDDs (C, DSDR > 30%, or D, CDRN ≥ 1). **(E, F)** At domain family level, domain families were defined using dominant category method; structural disorder of each species was measured by the percentage of intrinsically disordered domain families (E, family_DSDR > 30%, or F, family_CDRN ≥ 1). Scatter plots was made for organism complexity and structural disorder values obtained by different methods in fifty-one eukaryotes. Spearman method was used for the correlation analysis. The correlation coefficients (R) and P values (P) are shown in the inset, among which the significant results (P<0.01) are shown as red.

Figure S7. Box-plot of the percentage of intrinsically disordered (CDRN ≥ 1) domains (left panel) or domain families (right panel) in each species across the different phyla of eukaryotes. The four kingdoms of eukaryotes are marked on the bottom of the figure. The values of the upper and lower quartile, and the median values

are indicated as bars in the boxes. The differences in the percentage of disordered domain distribution between different categories are examined by Mann‑Whitney U test. The corrected *P* values are marked as stars in sub-figure G (*, $p < 0.05$; **, $p < 0.01$).

Figure S8. The distribution of IDDs in each age grade of the 25 representative species (related to figure 3C). Domains of each representative species are divided into different age grades. **A.** The distribution of IDDs (CDRN ≥ 1) in each age grade of the 25 representative species using SPOTD disorder degree results. **B, C.** The same analysis to figure 3C and A in this figure, using MobiDB-lite disorder degree results.

Figure S9. Comparison of the normalized DSDR values of the same domain families in evolutionarily more complex species and simpler species (related to figure 3D). Scatter-plot shows the median normalized DSDR values of the same domain families in complex species (top half of the figure) and simple species (bottom half of the figure). The red dots represent the difference of the median normalized DSDR value of each domain family between complex species and simple ones. The number and percentage of domain families belonging to each differential category are shown in brackets.

Figure S10. The number and functions of special IDDs in archaeal halobacteria. A. The difference between the IDDs in halobacterias and other archaea was shown in Venn diagram. B. Function analysis of the halobacterias-specific IDDs. C, D. Two examples of halobacterias-specific IDDs.

Figure S11. The special functions of IDDs in Ascomycota and Basidiomycota

revealed by the enriched mutant phenotypes. Over- or under-representation analysis of biological process (BP) and cellular component (CC) for the disordered domains. The values of ±log(P) were transformed into 14 grades (−7 to +7): −7, log(P) ≤ −8; −6, −8 < log(P) ≤ −6; −5, −6 < log(P) ≤ −4; −4, −4 < log(P) ≤ −2; −3, −2< log(P) ≤ −1.5; −2, −1.5 < log(P) ≤ −1.301; −0.25, −1.301 < log(P) ≤ 0; 0.25, 0 < −log(P) < 1.301; 2, 1.301 ≤ −log(P) <1.5; 3, 1.5≤ −log(P) < 2; 4, 2 ≤ −log(P) < 4; 5, 4 ≤ −log(P) < 6; 6, 6 ≤ −log(P) < 8; 7, −log(P) ≥ 8.

Figure S12. Over- or under-representation analysis of BPs for IDDs with different ages and different distribution width. The IDDs are divided into 6 categories. The over- or under-representation strengths of each class were represented by -log (P) or log (P), respectively. Heat map showing the grades of over- or under-representation strengths, scoped from -7 to 7 (See Method for detail).

Figure S13. The examples for the domains classified by domain age and disorder width (related to figure S12). The heatmaps show the normalized DSDR values of each domain in different clades. One protein instance for each domain was shown. The domain features, including domain age, disorder ratio, are at the bottom of each panel.

Figure S14. Comparison of the PTM site proportion in different type of domains/regions classified according to disorder degree. Box-plot of the PTM site proportion in protein domains with different disorder degree classified according to DSDR (A) or CDRN (B), or in CDR regions or other regions in domains (C). The values of upper and lower quartile, and the median values are indicated as bars in the boxes. The differences between the neighboring categories are examined by Mann-Whitney U

test. The corrected P values are shown. Six species were included in the analysis.

Figure S15. Scatter plot of the percentage of disordered domain families in each species of the three superkingdoms (related to figure 5A-C). Each point represents a species. The dashed diagonal line represents the line where Y and X are equal. The equations in each subfigure were obtained by fitting a straight line based on the data of all species of archaea (A), bacteria (B) and fungi or metazoan (C).

Figure S16. Scatter plot of the percentage of non-domain disordered regions in each species of the three superkingdoms (related to figure 5A-C). The N-terminal, C-terminal and linker regions in proteins were included in the analysis. See figure S7 for detailed descriptions.

Figure S17. The special features of the IDDs with a consecutive disorder region (CDR) but a low disorder ratio (DSDR<30%) (related to figure 5K). A. Comparison of the length between the class II and IV domains. B. Disorder prediction of transcriptional activator SEF1. C. The structure of a part of the sequence of SEF1, modeled by SWISS-MODEL. D. Disorder prediction of 3',5'-cyclic-nucleotide phosphodiesterase. E. The structure of 3',5'-cyclic-nucleotide phosphodiesterase modeled by SWISS-MODEL. The dashed lines in subfigure B and D represent the start and end positions of the protein domains and CDRs in them.

Figure S18. The distribution of IDDs and young domains in different repeating domain categories. Distribution of IDDs (A, B) and young domains (C) in different repeating domain categories of the twenty-five representative species. Dark spots represent statically significant differences of IDDs in the domains encoded only once

in the genome, tested by Fisher's exact test. Dark spots above the red dashed line represent that the domains encoded only once in the genome are over-represented compared to other types, while those below the blue dashed line represent they are under-represented. The abbreviations: *Hs, Homo sapiens; Sp, Strongylocentrotus purpuratus; Dm, Drosophila melanogaster; Cel, Caenorhabditis elegans; To, Thalassiosira oceanica; Em, Eimeria mitis; Eb, Enterocytozoon bieneusi; Up, Umbilicaria pustulata; Sc, Saccharomyces cerevisiae; At, Arabidopsis thaliana; Cr, Chlamydomonas reinhardtii; Ct, Chloroherpeton thalassium; Cex, Caldisericum exile; Ai, Alistipes inops; N, Nitrospina sp. SCGC_AAA799_A02; Ks, Kytococcus sedentarius; Td, Thermosulfurimonas dismutans; Ec, Escherichia coli; Lf, Leptospirillum ferriphilum; H, Hydrogenivirga sp. 128-5-R1-1; Cp, Chlamydia psittaci; Ht, Haloterrigena turkmenica; Kc, Candidatus korarchaeum cryptofilum; My, Metallosphaera yellowstonensis; No, Candidatus nitrocosmicus oleophilus*.

Figure S19. The method for the normalization of DSDR values and the classification of DSDR variation. A. Normalized curve for the DSDR values. Interpolation method was used to normalize the DSDRs. Four points were used to calculate the equation: (0, 0), (10, 1), (30, 2) and (100, 3). B. Grades of DSDR variation. The values denote the difference values of each pair of normalized DSDRs. C. Diagram of DSDR variation dominant category approach. Each domain pair has a DSDR variation value. We calculated the percentage of each DSDR variation grade and the dominant grade (> 0.5) was the DSDR variation dominant category of this domain family, which represented the level of variation of this domain family.

Figure S20. Correlation between the values of physico−chemical characteristics of amino acid and the values in P matrix. Physico−chemical characteristics of amino acids, such as hydropathy index (A), molecular weight (B) and isoelectric points (C) may influence the value of disorder score of each amino acid residue, then affect the values of DSDR. The cross represents the median of equal-sized bins. Whiskers encompass the range from a quarter to three quarters of values. The correlation coefficients (R) and *P* values (*P*) are shown in the inset.

Figure S21. The correlation between DSDR variation values and domain sequence similarity (A-D) and supplemental results about the distribution and functional features of domains with different DSDR variations (E-F). A-B. Correlation between DSDR variation and domain sequence similarity of each repeating domain pair. Scatter diagrams of human DSDR variation and domain sequence similarity in the domains repeating in the same proteins (A) or the domains repeating in multiple proteins (B). Each panel was divided into four regions according to the threshold low/high variation (DSDR variation = 1) and low/high similarity (40%). The bold numbers denote the numbers of domain pairs in each region. The abbreviations: LS, low similarity; HS, high similarity; LV, low variation; HV, high variation. **C-D**. Heatmap shows the numbers and percentages of four types of domain pairs in the 25 representative species. The percentages of low-similarity domain pairs (LS/Total) and the percentages of low-DSDR variation domain pairs among the low-similarity domain pairs (LS&LV/LS) in each species are listed on the right of the heat map. Sub-figure C shows for the domains repeating in the same proteins and D shows the domains

repeating in multiple proteins. **E.** Distribution of No-variation dominant categories of DSDR variation in the domains repeating in the same proteins and the domains appearing among multiple proteins of the 25 representative species using MobiDB-lite disorder result. The DSDR variation value was calculated by the difference of normalized DSDR. Black spots represent statically significant differences, tested by the Fisher's exact test. Black spots above the dashed line represent significant over representations. The abbreviations: *Hs, Homo sapiens; Sp, Strongylocentrotus purpuratus; Dm, Drosophila melanogaster; Cel, Caenorhabditis elegans; Eca, Enterospora canceri; Lp, Lasallia pustulata; Sc, Saccharomyces cerevisiae; To, Thalassiosira oceanica; Cc, Cyclospora cayetanensis; At, Arabidopsis thaliana; Cr, Chlamydomonas reinhardtii; Ct, Chloroherpeton thalassium; Cex, Caldisericum exile; Ai, Alistipes inops; N, Nitrospina sp. SCGC_AAA799_A02; Ks, Kytococcus sedentarius; Td, Thermosulfurimonas dismutans; Ec, Escherichia coli; Lf, Leptospirillum ferriphilum; H, Hydrogenivirga sp. 128-5-R1-1; Cp, Chlamydia psittaci; Hd, Haloterrigena daqingensis; Kc, Candidatus korarchaeum cryptofilum; My, Metallosphaera yellowstonensis; No, Candidatus nitrocosmicus oleophilus.* **F.** Examples of 'no-variation' domains. DSDR was normalized by interpolation method. Heatmap shows the normalized DSDR values of the same domain family in different proteins of different species. Each column represents a different species, and each row represents the corresponding orthologs. The abbreviations of the species: *Han, Helianthus annuus; Gs, Galdieria sulphuraria; Cc, Chondrus crispus; Cm,*

Cyanidioschyzon merolae; Dc, Daucus carota; Atr, Amborella trichopoda; At, Arabidopsis thaliana; Bn, Brassica napus; Mt, Medicago truncatula.

Figure S22. Comparison of domain length among the protein domains with different disorder degrees. Protein domains were classified according to CDRN (A-C) or DSDR (D-F) grades. The protein domains from archaea (A, D), bacteria (B, E) and eukaryotes (C, F) were analyzed respectively. The values of upper and lower quartile are indicated as upper and lower edges of the box, and the median values are indicated as a bar in the box. The differences in the domain length between different categories are examined by Mann–Whitney U test. The corrected *P* values are shown at the top of each panel.

Figure S1

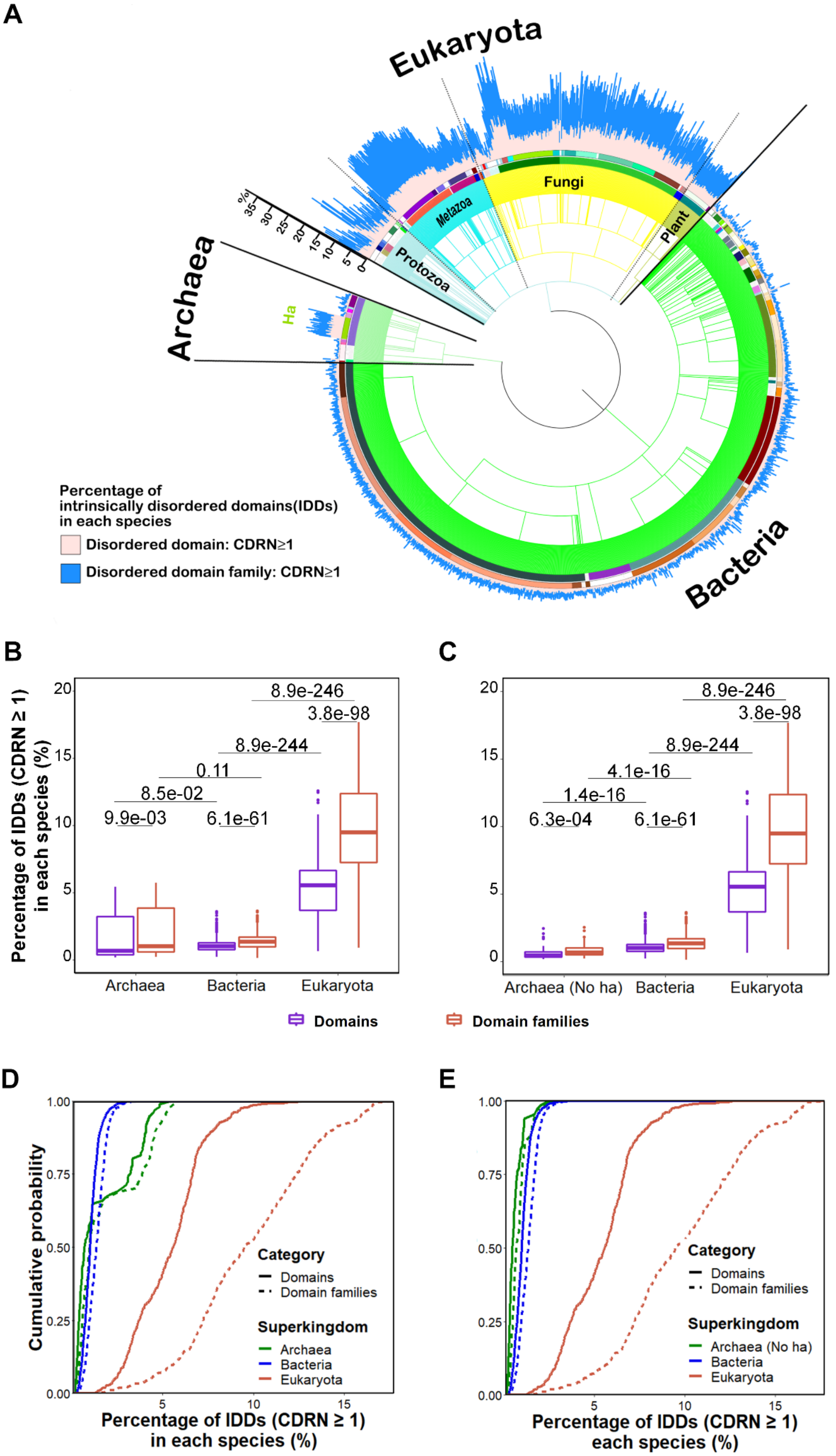
A
Eukaryota
Archaea
Bacteria
Fungi
Metazoa
Protozoa
Plant
Ha
%
35
30
25
20
15
10
5
0
Percentage of intrinsically disordered domains(IDDs) in each species
Disordered domain: CDRN≥1
Disordered domain family: CDRN≥1
B
C
Percentage of IDDs (CDRN ≥ 1) in each species (%)
20
15
10
5
0
8.9e-246
3.8e-98
8.9e-244
0.11
8.5e-02
9.9e-03
6.1e-61
4.1e-16
1.4e-16
6.3e-04
Archaea
Bacteria
Eukaryota
Archaea (No ha)
Domains
Domain families
D
E
Cumulative probability
1.00
0.75
0.50
0.25
0.00
Category
Domains
Domain families
Superkingdom
Archaea
Bacteria
Eukaryota
Archaea (No ha)
Percentage of IDDs (CDRN ≥ 1) in each species (%)
Percentage of IDDs (CDRN ≥ 1) each species (%)

Figure S2

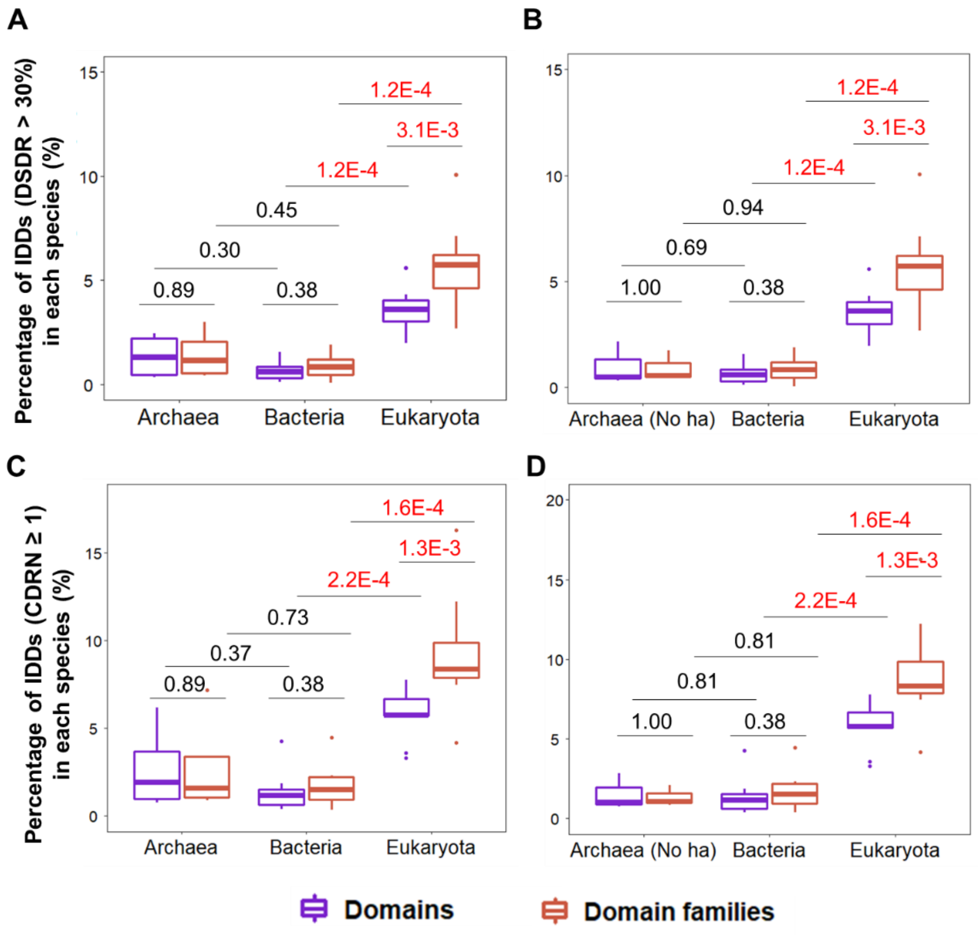

A
B
C
D
Percentage of IDDs (DSDR > 30%) in each species (%)
Percentage of IDDs (CDRN ≥ 1) in each species (%)
1.2E-4
3.1E-3
1.2E-4
0.45
0.30
0.89
0.38
0.94
0.69
1.00
1.6E-4
1.3E-3
2.2E-4
0.73
0.37
0.81
Archaea
Bacteria
Eukaryota
Archaea (No ha)
Domains
Domain families

Figure S3

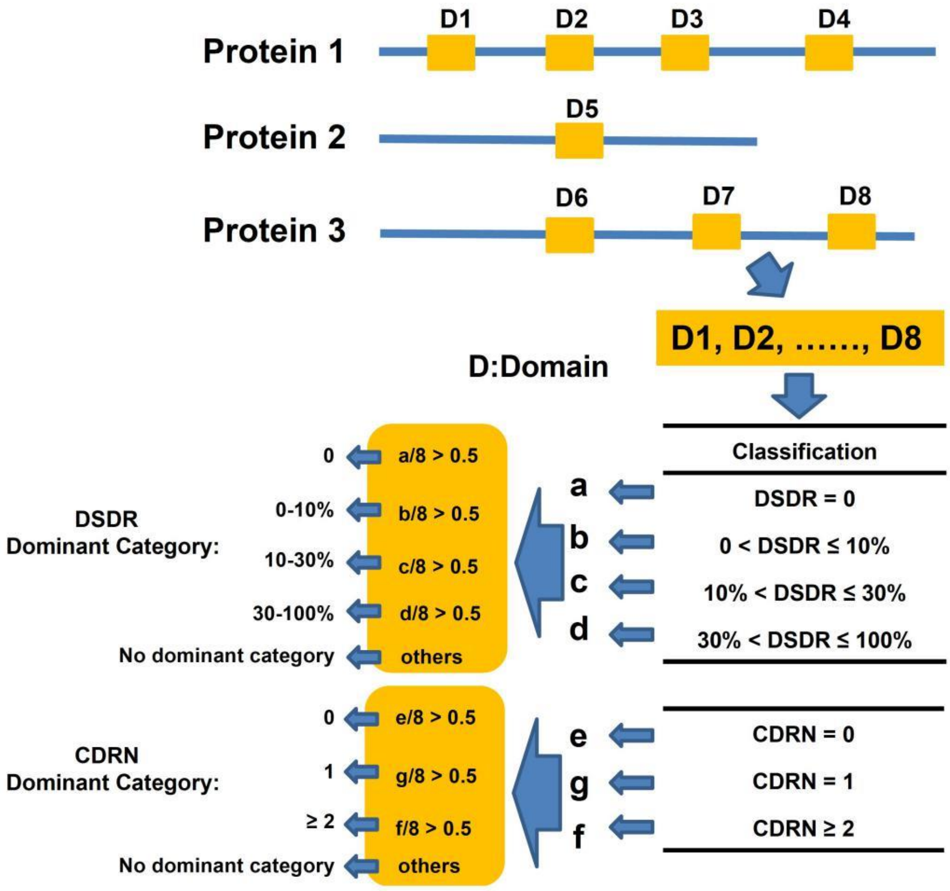

Protein 1
D1
D2
D3
D4
Protein 2
D5
Protein 3
D6
D7
D8
D1, D2, ……, D8
D:Domain
Classification
a
DSDR = 0
b
0 < DSDR ≤ 10%
c
10% < DSDR ≤ 30%
d
30% < DSDR ≤ 100%
e
CDRN = 0
g
CDRN = 1
f
CDRN ≥ 2
DSDR
Dominant Category:
0
a/8 > 0.5
0-10%
b/8 > 0.5
10-30%
c/8 > 0.5
30-100%
d/8 > 0.5
No dominant category
others
CDRN
Dominant Category:
0
e/8 > 0.5
1
g/8 > 0.5
≥ 2
f/8 > 0.5
No dominant category
others

Figure S4

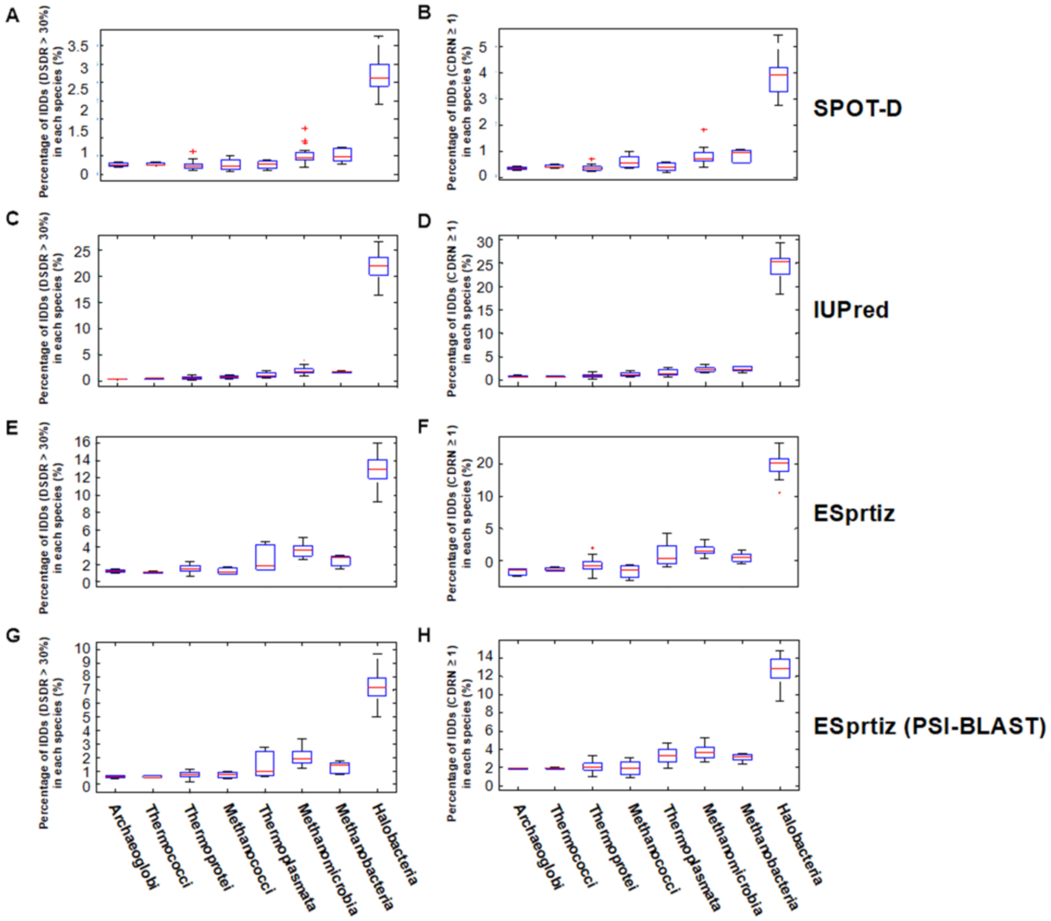

A
B
C
D
E
F
G
H
SPOT-D
IUPred
ESprtiz
ESprtiz (PSI-BLAST)
Percentage of IDDs (DSDR > 30%) in each species (%)
Percentage of IDDs (CDRN ≥ 1) in each species (%)
Archaeoglobi
Thermococci
Thermoprotei
Methanococci
Thermoplasmata
Methanomicrobia
Methanobacteria
Halobacteria

Figure S5

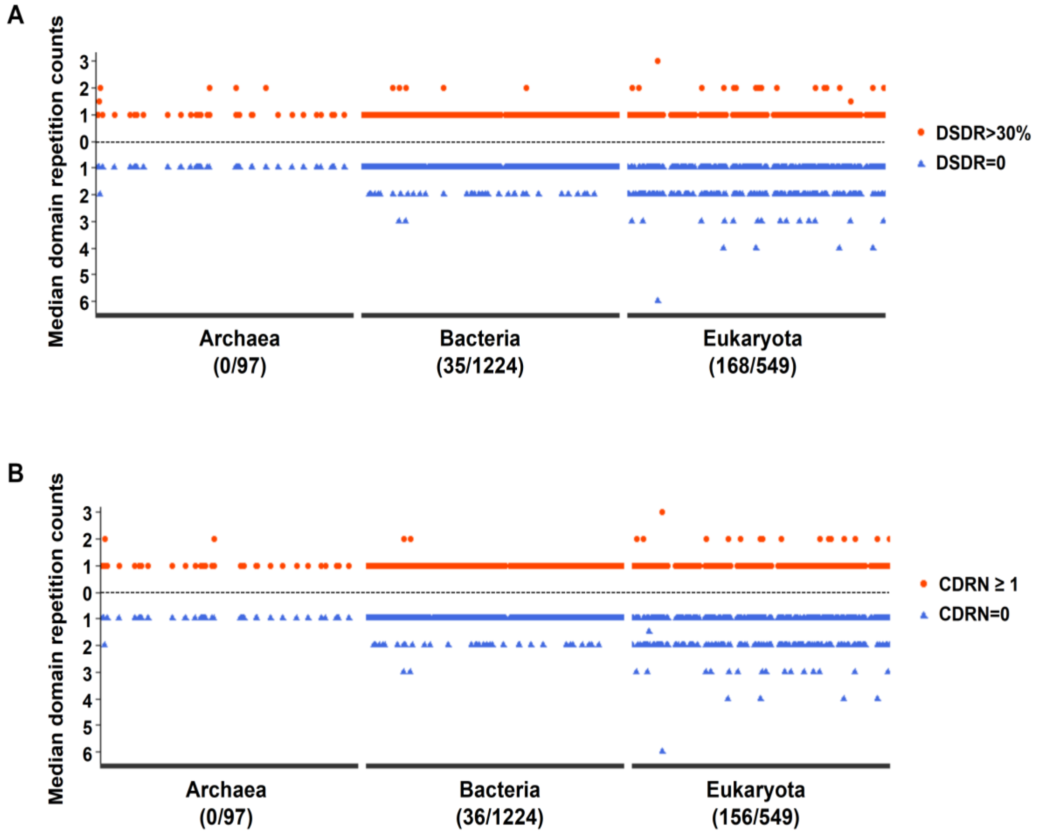

A
Median domain repetition counts
3
2
1
0
1
2
3
4
5
6
Archaea
(0/97)
Bacteria
(35/1224)
Eukaryota
(168/549)
DSDR>30%
DSDR=0
B
Median domain repetition counts
3
2
1
0
1
2
3
4
5
6
Archaea
(0/97)
Bacteria
(36/1224)
Eukaryota
(156/549)
CDRN ≥ 1
CDRN=0

Figure S6

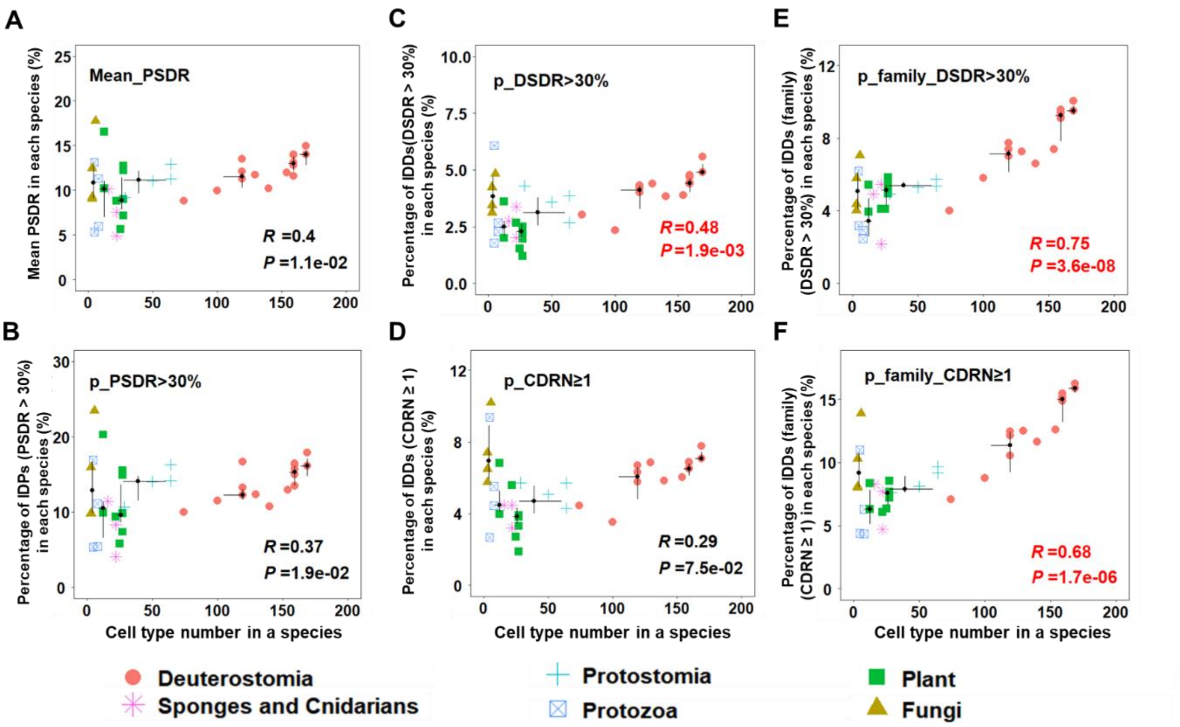

A
Mean_PSDR
Mean PSDR in each species (%)
R =0.4
P =1.1e-02
B
p_PSDR>30%
Percentage of IDPs (PSDR > 30%) in each species (%)
R =0.37
P =1.9e-02
Cell type number in a species
C
p_DSDR>30%
Percentage of IDDs(DSDR > 30%) in each species (%)
R =0.48
P =1.9e-03
D
p_CDRN≥1
Percentage of IDDs (CDRN ≥ 1) in each species (%)
R =0.29
P =7.5e-02
Cell type number in a species
E
p_family_DSDR>30%
Percentage of IDDs (family) (DSDR > 30%) in each species (%)
R =0.75
P =3.6e-08
F
p_family_CDRN≥1
Percentage of IDDs (family) (CDRN ≥ 1) in each species (%)
R =0.68
P =1.7e-06
Cell type number in a species
Deuterostomia
Sponges and Cnidarians
Protostomia
Protozoa
Plant
Fungi

Figure S7

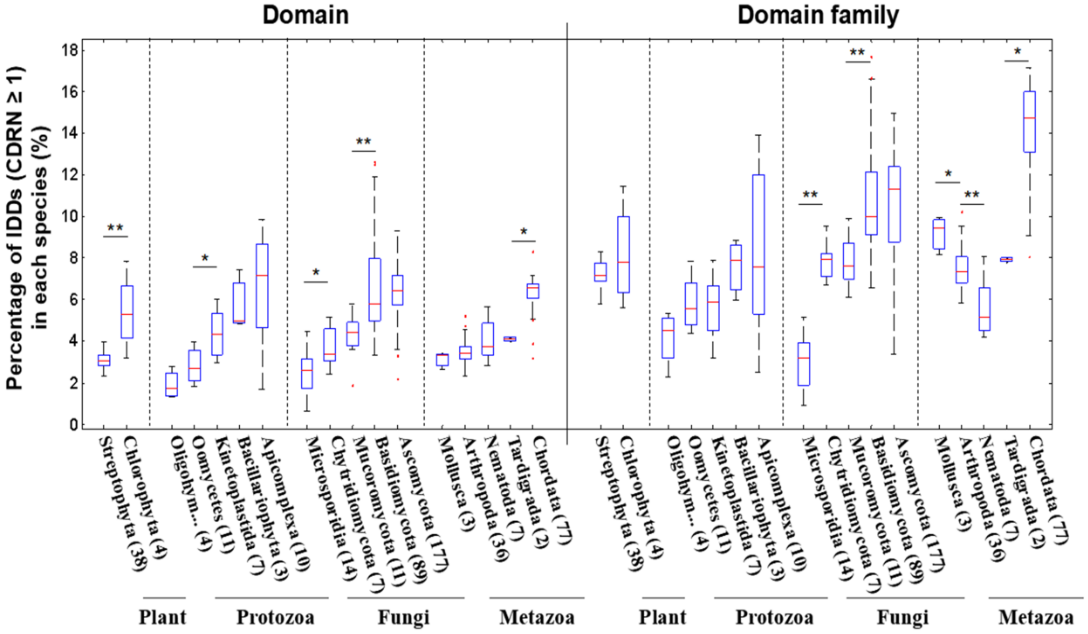

Domain
Domain family
Percentage of IDDs (CDRN ≥ 1) in each species (%)
Streptophyta (38)
Chlorophyta (4)
Oligohym... (4)
Oomycetes (11)
Kinetoplastida (7)
Bacillariophyta (3)
Apicomplexa (10)
Microsporidia (14)
Chytridiomycota (7)
Mucoromycota (11)
Basidiomycota (89)
Ascomycota (177)
Mollusca (3)
Arthropoda (36)
Nematoda (7)
Tardigrada (2)
Chordata (77)
Plant
Protozoa
Fungi
Metazoa

Figure S8

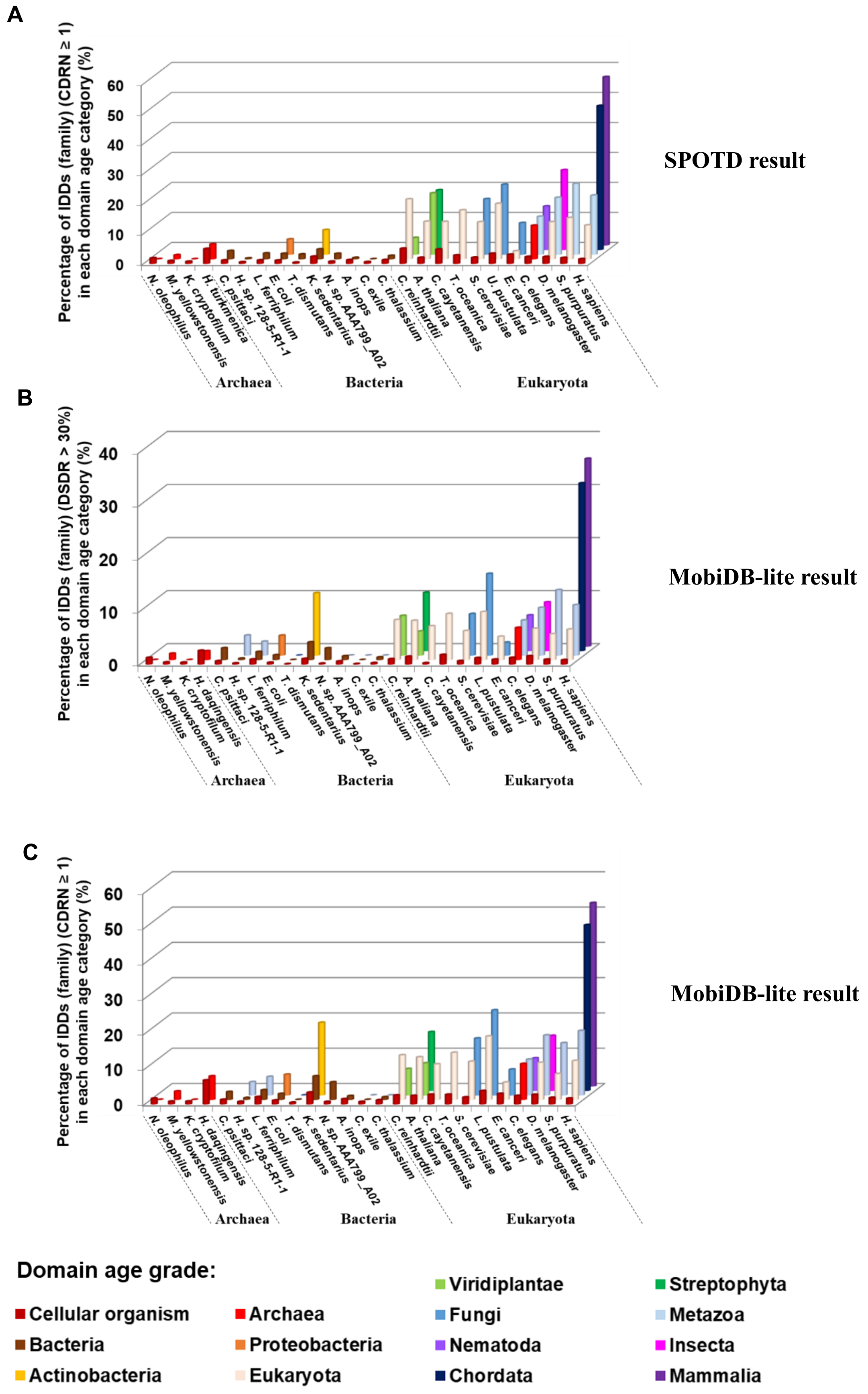
A
Percentage of IDDs (family) (CDRN ≥ 1) in each domain age category (%)
SPOTD result
Archaea
Bacteria
Eukaryota
B
Percentage of IDDs (family) (DSDR > 30%) in each domain age category (%)
MobiDB-lite result
C
Percentage of IDDs (family) (CDRN ≥ 1) in each domain age category (%)
MobiDB-lite result
Domain age grade:
Cellular organism
Archaea
Bacteria
Proteobacteria
Actinobacteria
Eukaryota
Viridiplantae
Fungi
Nematoda
Chordata
Streptophyta
Metazoa
Insecta
Mammalia

Figure S9

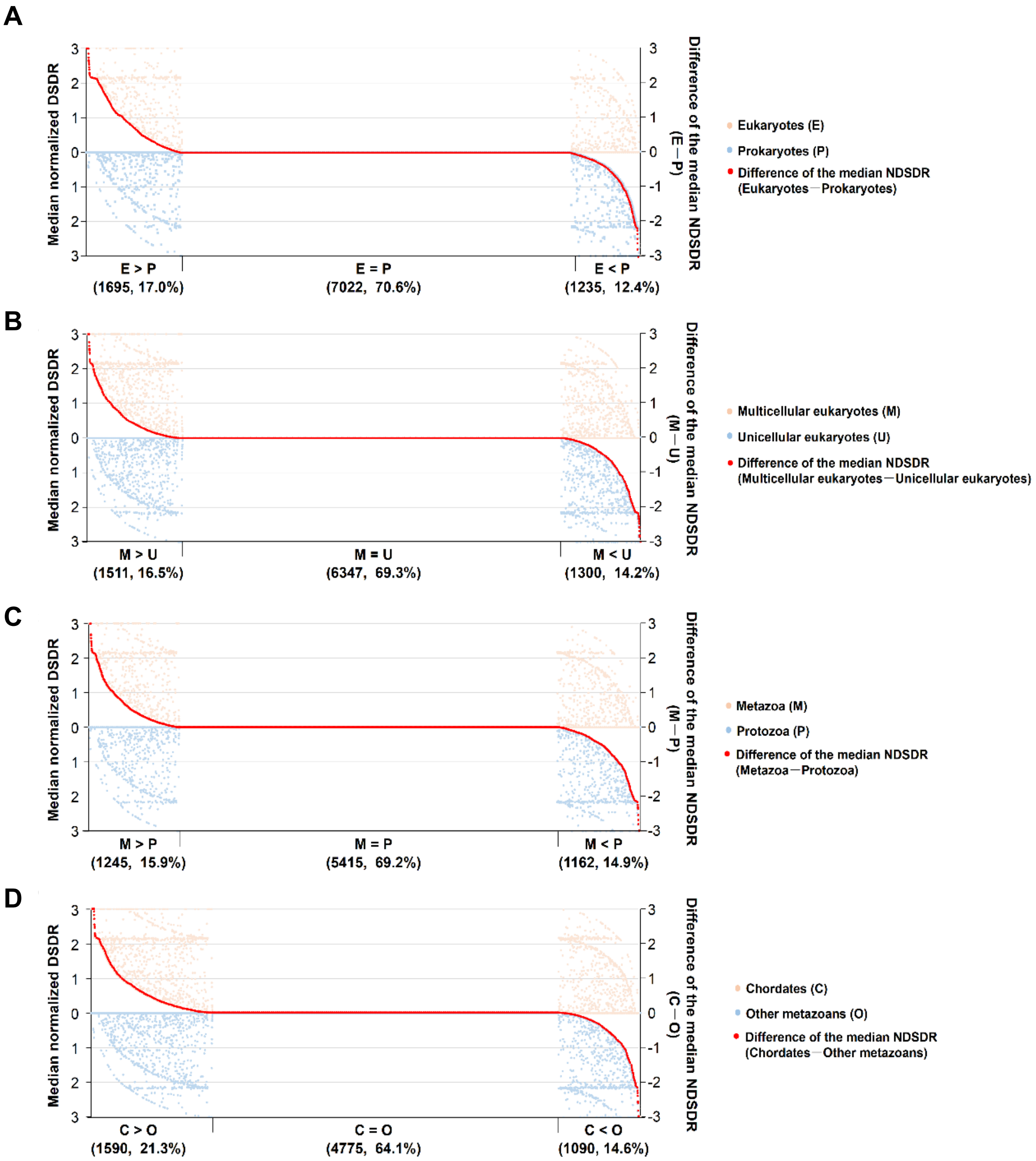
A
Median normalized DSDR
Difference of the median NDSDR (E − P)
E > P
(1695, 17.0%)
E = P
(7022, 70.6%)
E < P
(1235, 12.4%)
Eukaryotes (E)
Prokaryotes (P)
Difference of the median NDSDR (Eukaryotes − Prokaryotes)
B
Median normalized DSDR
Difference of the median NDSDR (M − U)
M > U
(1511, 16.5%)
M = U
(6347, 69.3%)
M < U
(1300, 14.2%)
Multicellular eukaryotes (M)
Unicellular eukaryotes (U)
Difference of the median NDSDR (Multicellular eukaryotes − Unicellular eukaryotes)
C
Median normalized DSDR
Difference of the median NDSDR (M − P)
M > P
(1245, 15.9%)
M = P
(5415, 69.2%)
M < P
(1162, 14.9%)
Metazoa (M)
Protozoa (P)
Difference of the median NDSDR (Metazoa − Protozoa)
D
Median normalized DSDR
Difference of the median NDSDR (C − O)
C > O
(1590, 21.3%)
C = O
(4775, 64.1%)
C < O
(1090, 14.6%)
Chordates (C)
Other metazoans (O)
Difference of the median NDSDR (Chordates − Other metazoans)

Figure S10

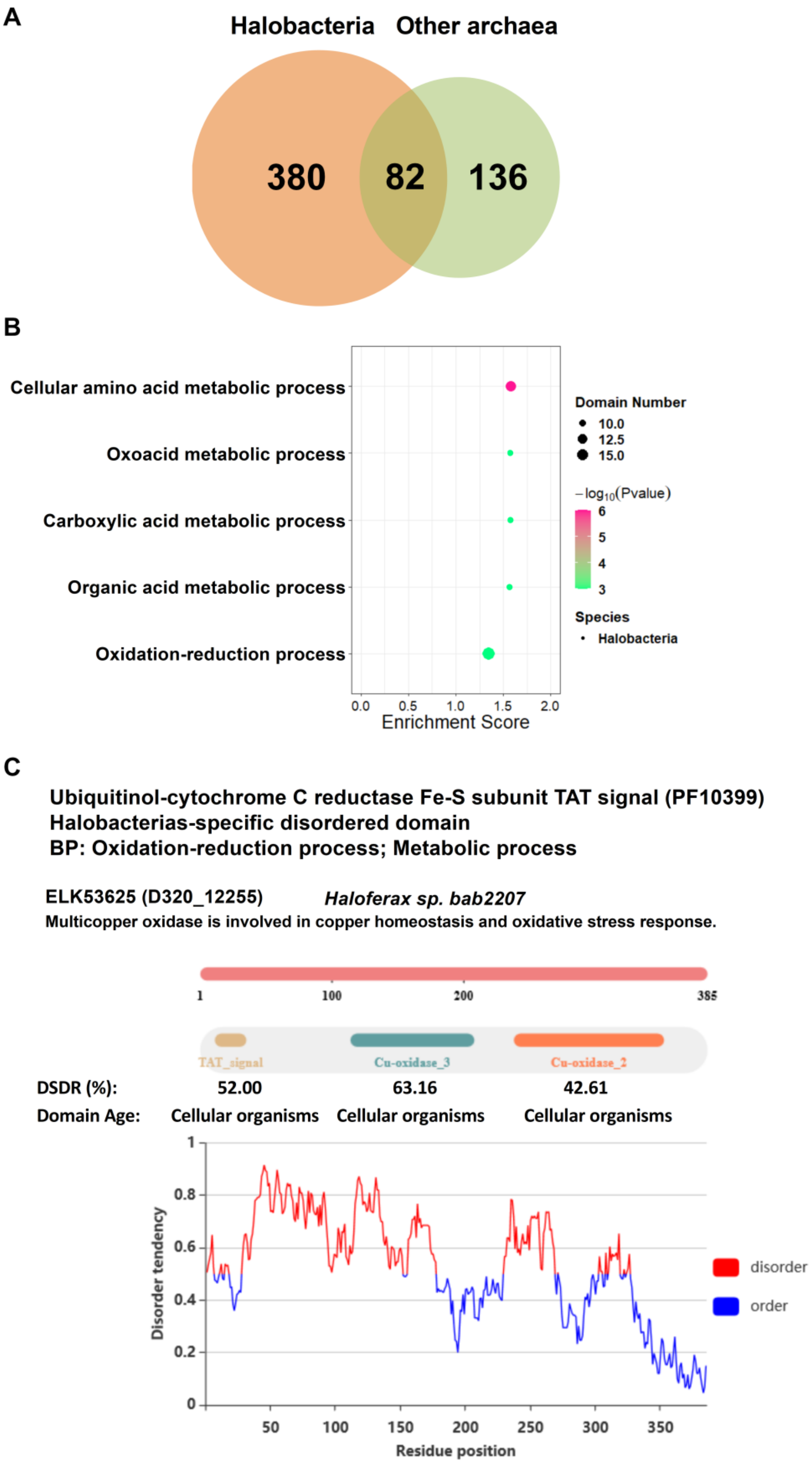
A
Halobacteria
Other archaea
380
82
136
B
Cellular amino acid metabolic process
Oxoacid metabolic process
Carboxylic acid metabolic process
Organic acid metabolic process
Oxidation-reduction process
0.0
0.5
1.0
1.5
2.0
Enrichment Score
Domain Number
10.0
12.5
15.0
−log10(Pvalue)
6
5
4
3
Species
Halobacteria
C
Ubiquitinol-cytochrome C reductase Fe-S subunit TAT signal (PF10399)
Halobacterias-specific disordered domain
BP: Oxidation-reduction process; Metabolic process
ELK53625 (D320_12255)
Haloferax sp. bab2207
Multicopper oxidase is involved in copper homeostasis and oxidative stress response.
1
100
200
385
TAT_signal
Cu-oxidase_3
Cu-oxidase_2
DSDR (%):
52.00
63.16
42.61
Domain Age:
Cellular organisms
Cellular organisms
Cellular organisms
Disorder tendency
1
0.8
0.6
0.4
0.2
0
50
100
150
200
250
300
350
Residue position
disorder
order

Figure S11

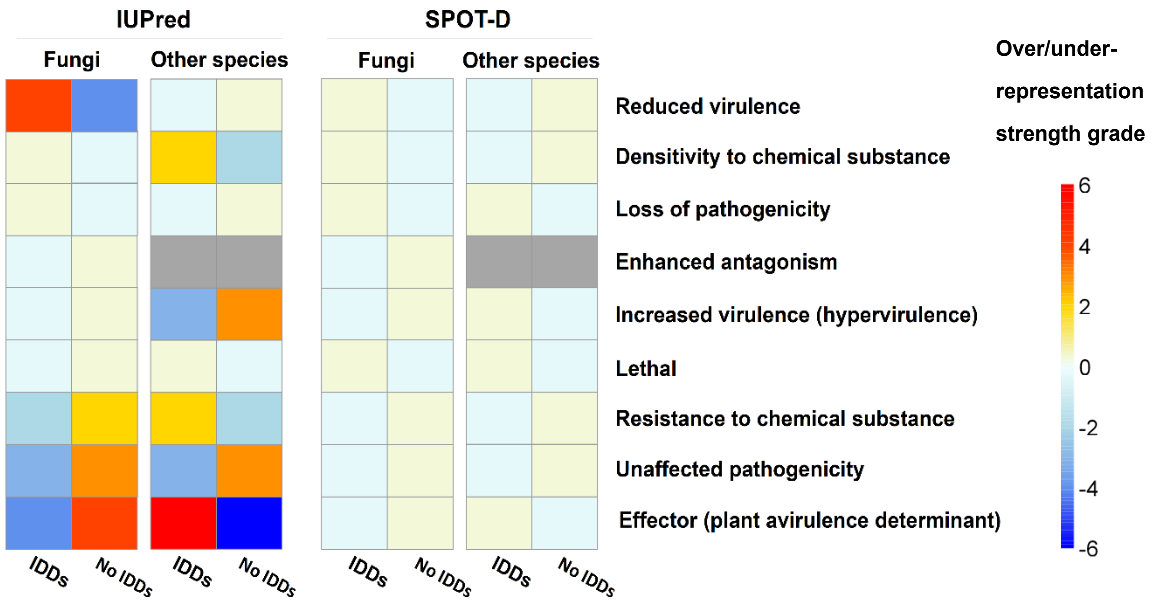
IUPred
SPOT-D
Fungi
Other species
Fungi
Other species
Reduced virulence
Densitivity to chemical substance
Loss of pathogenicity
Enhanced antagonism
Increased virulence (hypervirulence)
Lethal
Resistance to chemical substance
Unaffected pathogenicity
Effector (plant avirulence determinant)
IDDs
No IDDs
IDDs
No IDDs
IDDs
No IDDs
IDDs
No IDDs
Over/under-
representation
strength grade
6
4
2
0
-2
-4
-6

Figure S12

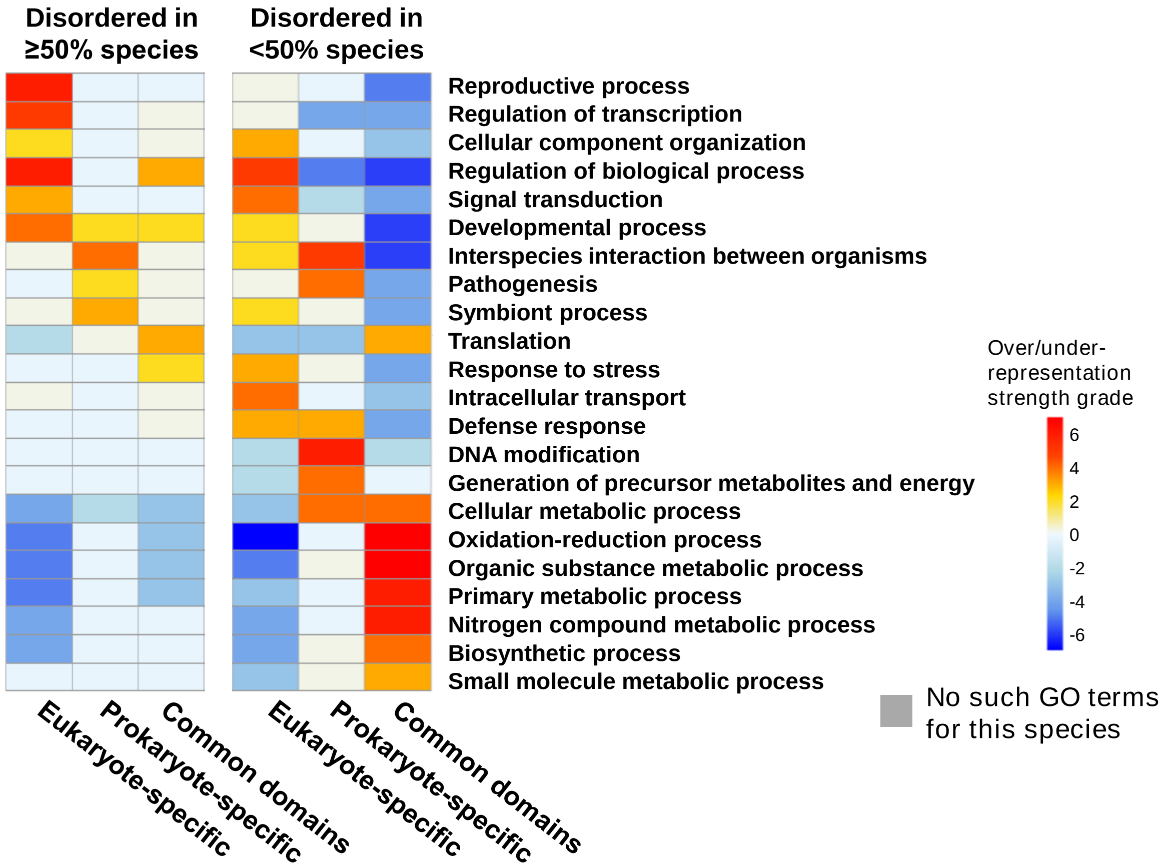

Disordered in ≥50% species
Disordered in <50% species
Reproductive process
Regulation of transcription
Cellular component organization
Regulation of biological process
Signal transduction
Developmental process
Interspecies interaction between organisms
Pathogenesis
Symbiont process
Translation
Response to stress
Intracellular transport
Defense response
DNA modification
Generation of precursor metabolites and energy
Cellular metabolic process
Oxidation-reduction process
Organic substance metabolic process
Primary metabolic process
Nitrogen compound metabolic process
Biosynthetic process
Small molecule metabolic process
Eukaryote-specific
Prokaryote-specific
Common domains
Eukaryote-specific
Prokaryote-specific
Common domains
Over/under-representation strength grade
6
4
2
0
-2
-4
-6
No such GO terms for this species

Figure S13

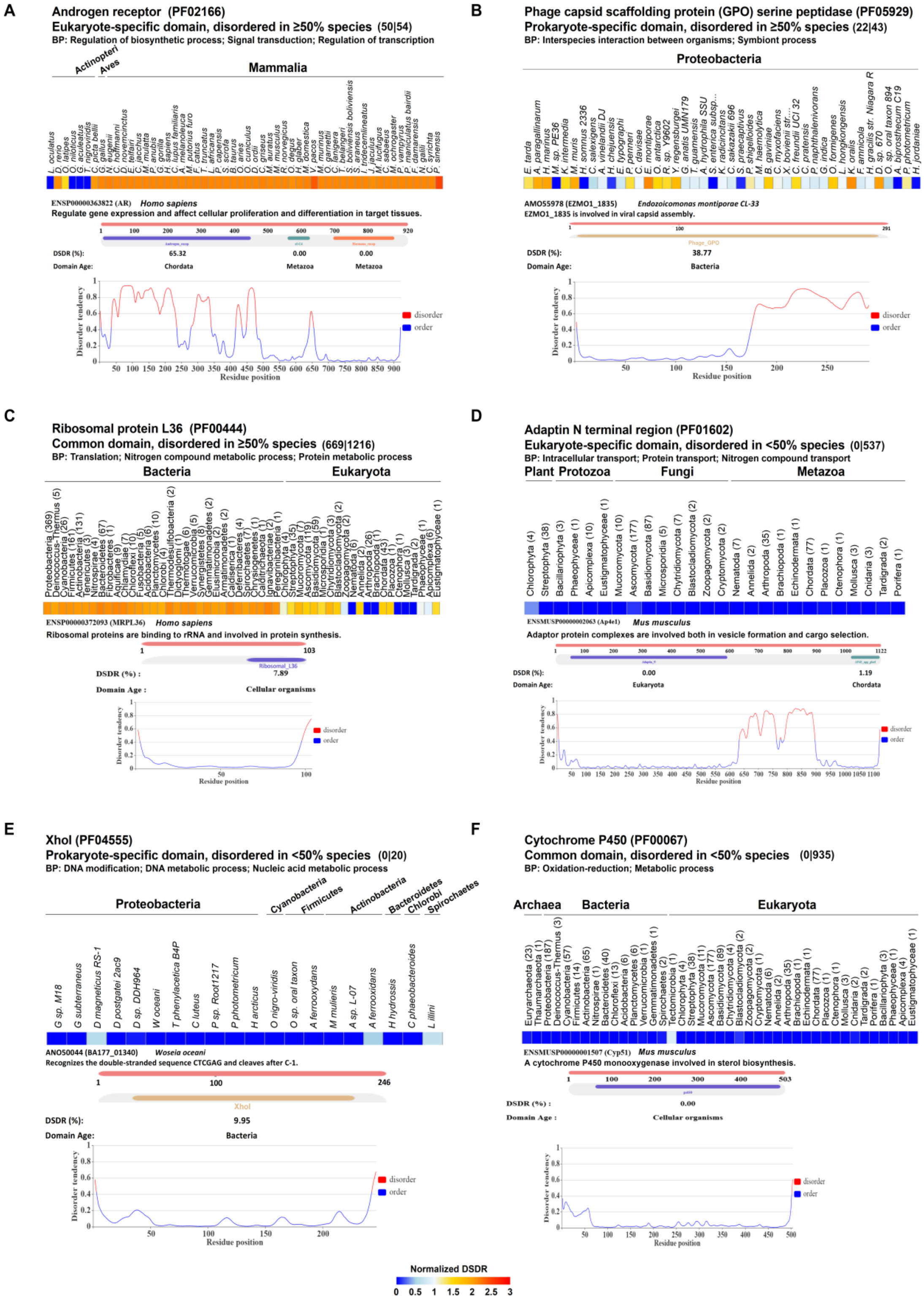
A
Androgen receptor (PF02166)
Eukaryote-specific domain, disordered in ≥50% species (50|54)
BP: Regulation of biosynthetic process; Signal transduction; Regulation of transcription
Actinopteri
Aves
Mammalia
ENSP00000363822 (AR) Homo sapiens
Regulate gene expression and affect cellular proliferation and differentiation in target tissues.
DSDR (%): 65.32 0.00 0.00
Domain Age: Chordata Metazoa Metazoa
Disorder tendency
Residue position
disorder
order
B
Phage capsid scaffolding protein (GPO) serine peptidase (PF05929)
Prokaryote-specific domain, disordered in ≥50% species (22|43)
BP: Interspecies interaction between organisms; Symbiont process
Proteobacteria
AMO55978 (EZMO1_1835) Endozoicomonas montiporae CL-33
EZMO1_1835 is involved in viral capsid assembly.
Phage_GPO
DSDR (%): 38.77
Domain Age: Bacteria
C
Ribosomal protein L36 (PF00444)
Common domain, disordered in ≥50% species (669|1216)
BP: Translation; Nitrogen compound metabolic process; Protein metabolic process
Bacteria
Eukaryota
ENSP00000372093 (MRPL36) Homo sapiens
Ribosomal proteins are binding to rRNA and involved in protein synthesis.
Ribosomal_L36
DSDR (%) : 7.89
Domain Age : Cellular organisms
D
Adaptin N terminal region (PF01602)
Eukaryote-specific domain, disordered in <50% species (0|537)
BP: Intracellular transport; Protein transport; Nitrogen compound transport
Plant
Protozoa
Fungi
Metazoa
Chlorophyta (4)
Streptophyta (38)
Bacillariophyta (3)
Phaeophyceae (1)
Apicomplexa (10)
Eustigmatophyceae (1)
Mucoromycota (10)
Ascomycota (177)
Basidiomycota (87)
Microsporidia (5)
Chytridiomycota (7)
Blastocladiomycota (2)
Zoopagomycota (2)
Cryptomycota (2)
Nematoda (7)
Annelida (2)
Arthropoda (35)
Brachiopoda (1)
Echinodermata (1)
Chordata (77)
Placozoa (1)
Ctenophora (1)
Mollusca (3)
Cnidaria (3)
Tardigrada (2)
Porifera (1)
ENSMUSP00000002063 (Ap4e1) Mus musculus
Adaptor protein complexes are involved both in vesicle formation and cargo selection.
DSDR (%): 0.00 1.19
Domain Age: Eukaryota Chordata
E
XhoI (PF04555)
Prokaryote-specific domain, disordered in <50% species (0|20)
BP: DNA modification; DNA metabolic process; Nucleic acid metabolic process
Proteobacteria
Cyanobacteria
Firmicutes
Actinobacteria
Bacteroidetes
Chlorobi
Spirochaetes
ANO50044 (BA177_01340) Woseia oceani
Recognizes the double-stranded sequence CTCGAG and cleaves after C-1.
XhoI
DSDR (%): 9.95
Domain Age: Bacteria
F
Cytochrome P450 (PF00067)
Common domain, disordered in <50% species (0|935)
BP: Oxidation-reduction; Metabolic process
Archaea
Bacteria
Eukaryota
ENSMUSP00000001507 (Cyp51) Mus musculus
A cytochrome P450 monooxygenase involved in sterol biosynthesis.
DSDR (%) : 0.00
Domain Age : Cellular organisms
Normalized DSDR
0 0.5 1 1.5 2 2.5 3

Figure S14

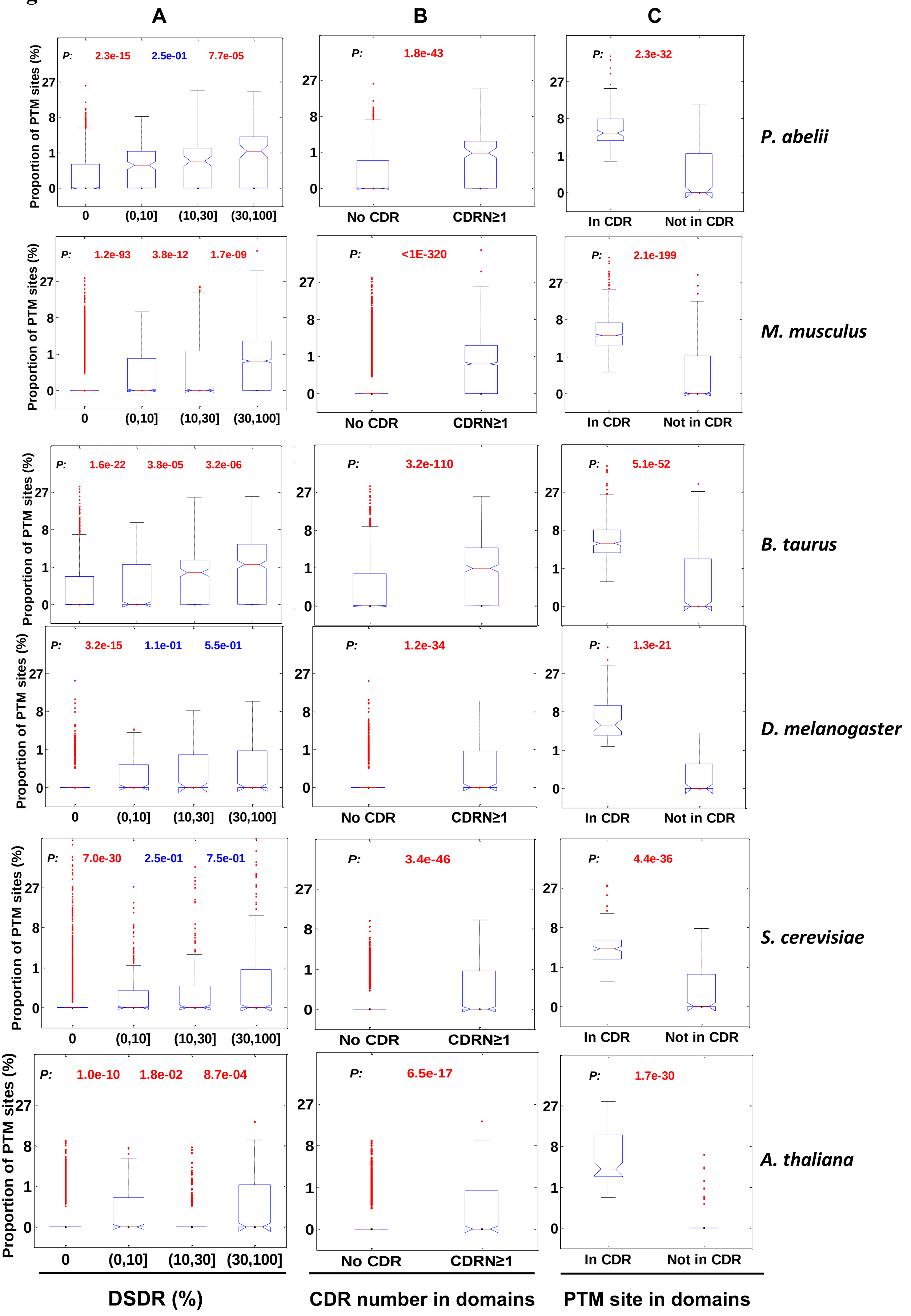
A
B
C
Proportion of PTM sites (%)
P: 2.3e-15 2.5e-01 7.7e-05
P: 1.8e-43
P: 2.3e-32
P. abelii
P: 1.2e-93 3.8e-12 1.7e-09
P: <1E-320
P: 2.1e-199
M. musculus
P: 1.6e-22 3.8e-05 3.2e-06
P: 3.2e-110
P: 5.1e-52
B. taurus
P: 3.2e-15 1.1e-01 5.5e-01
P: 1.2e-34
P: 1.3e-21
D. melanogaster
P: 7.0e-30 2.5e-01 7.5e-01
P: 3.4e-46
P: 4.4e-36
S. cerevisiae
P: 1.0e-10 1.8e-02 8.7e-04
P: 6.5e-17
P: 1.7e-30
A. thaliana
0 1 8 27
0 (0,10] (10,30] (30,100]
No CDR CDRN≥1
In CDR Not in CDR
DSDR (%)
CDR number in domains
PTM site in domains

Figure S15

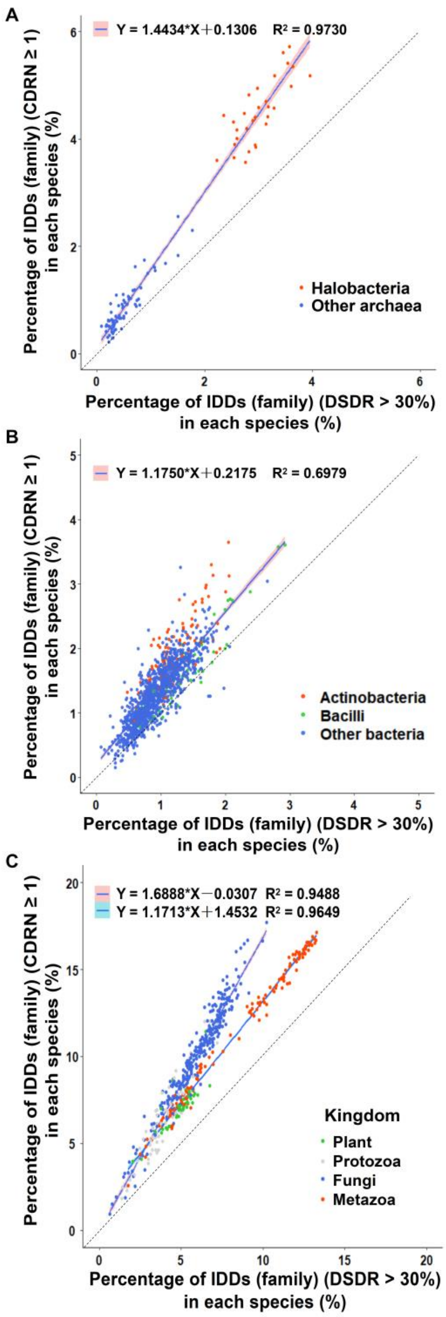
A
Y = 1.4434*X＋0.1306 R² = 0.9730
Halobacteria
Other archaea
Percentage of IDDs (family) (CDRN ≥ 1) in each species (%)
Percentage of IDDs (family) (DSDR > 30%) in each species (%)
B
Y = 1.1750*X＋0.2175 R² = 0.6979
Actinobacteria
Bacilli
Other bacteria
Percentage of IDDs (family) (CDRN ≥ 1) in each species (%)
Percentage of IDDs (family) (DSDR > 30%) in each species (%)
C
Y = 1.6888*X－0.0307 R² = 0.9488
Y = 1.1713*X＋1.4532 R² = 0.9649
Kingdom
Plant
Protozoa
Fungi
Metazoa
Percentage of IDDs (family) (CDRN ≥ 1) in each species (%)
Percentage of IDDs (family) (DSDR > 30%) in each species (%)

Figure S16

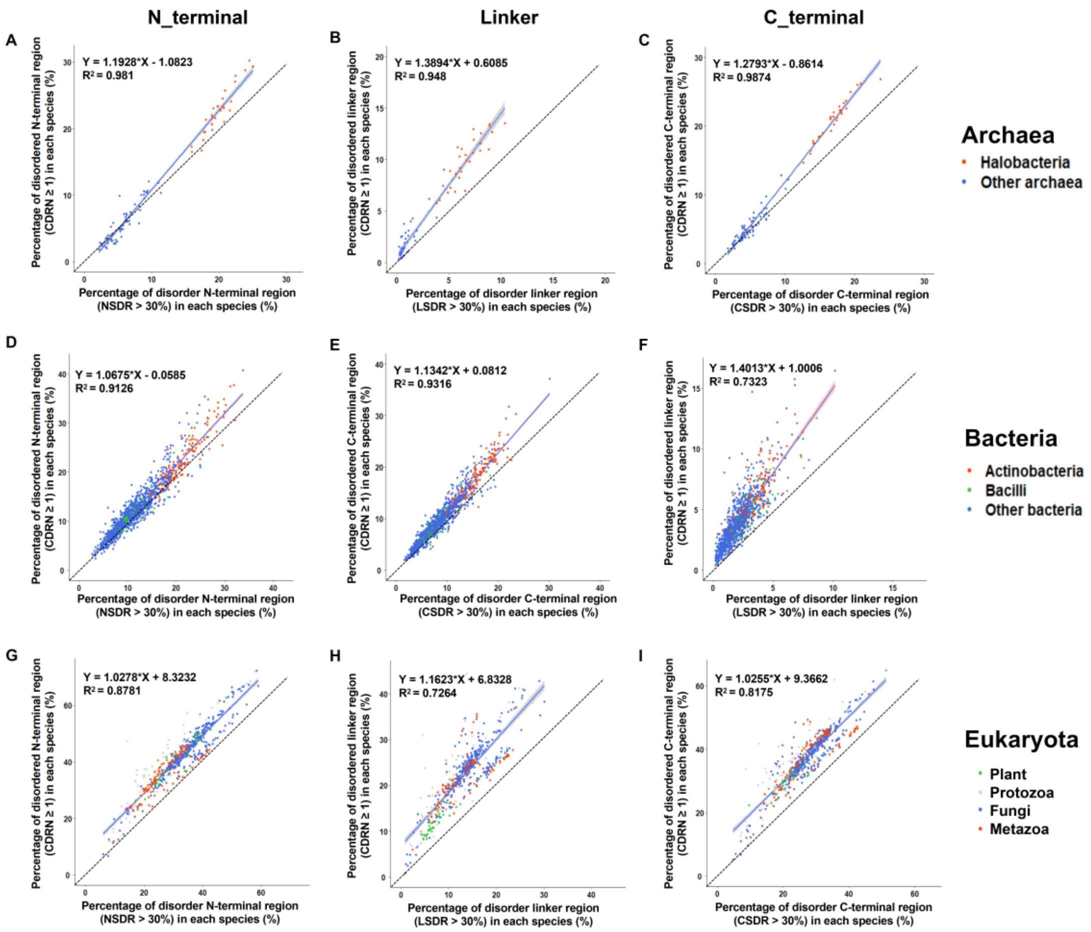

N_terminal
Linker
C_terminal
A
Y = 1.1928*X - 1.0823
R² = 0.981
B
Y = 1.3894*X + 0.6085
R² = 0.948
C
Y = 1.2793*X - 0.8614
R² = 0.9874
Archaea
Halobacteria
Other archaea
D
Y = 1.0675*X - 0.0585
R² = 0.9126
E
Y = 1.1342*X + 0.0812
R² = 0.9316
F
Y = 1.4013*X + 1.0006
R² = 0.7323
Bacteria
Actinobacteria
Bacilli
Other bacteria
G
Y = 1.0278*X + 8.3232
R² = 0.8781
H
Y = 1.1623*X + 6.8328
R² = 0.7264
I
Y = 1.0255*X + 9.3662
R² = 0.8175
Eukaryota
Plant
Protozoa
Fungi
Metazoa
Percentage of disordered N-terminal region (CDRN ≥ 1) in each species (%)
Percentage of disordered linker region (CDRN ≥ 1) in each species (%)
Percentage of disordered C-terminal region (CDRN ≥ 1) in each species (%)
Percentage of disorder N-terminal region (NSDR > 30%) in each species (%)
Percentage of disorder linker region (LSDR > 30%) in each species (%)
Percentage of disorder C-terminal region (CSDR > 30%) in each species (%)

Figure S17

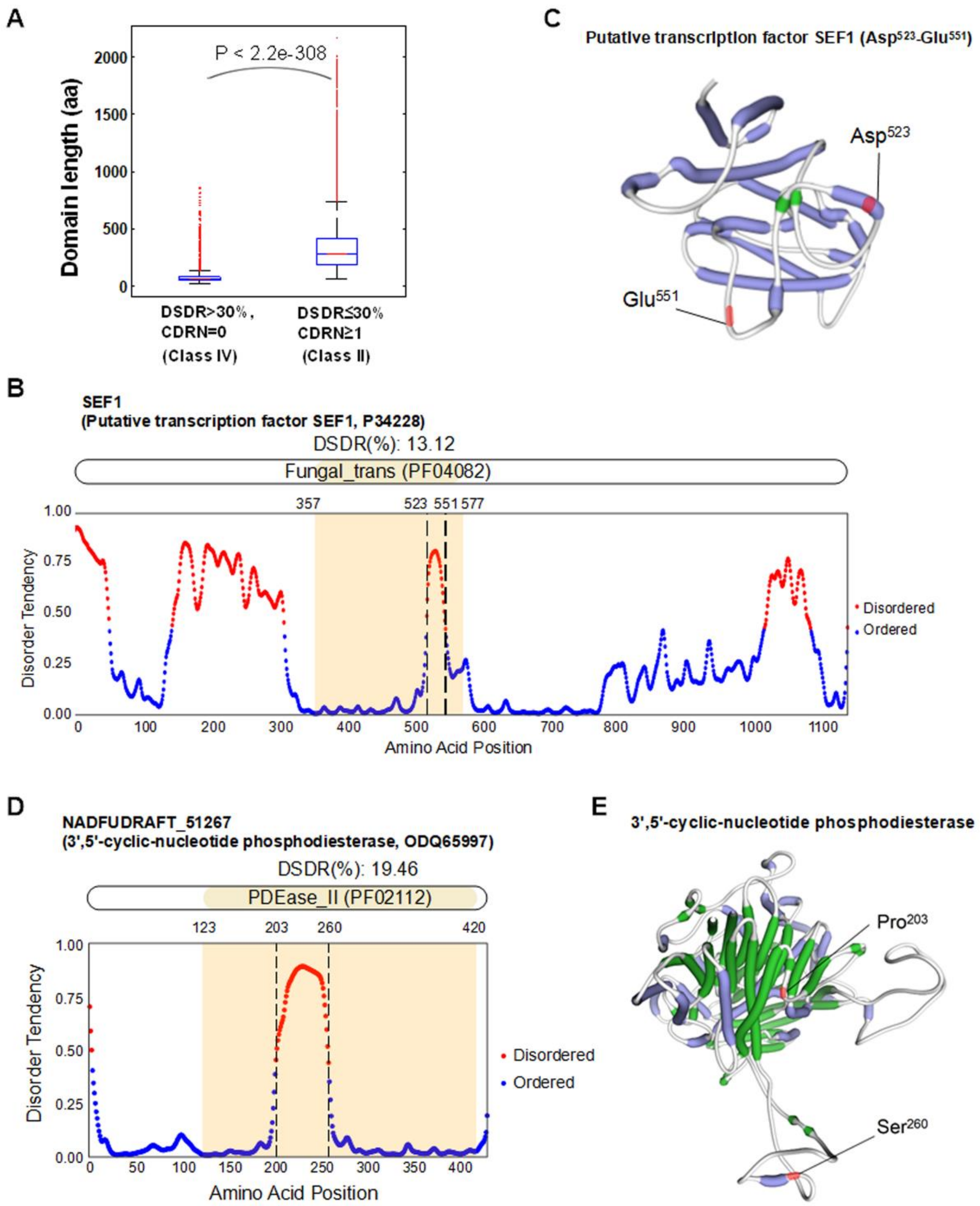
A
P < 2.2e-308
Domain length (aa)
2000
1500
1000
500
0
DSDR>30%,
CDRN=0
(Class IV)
DSDR≤30%
CDRN≥1
(Class II)
B
SEF1
(Putative transcription factor SEF1, P34228)
DSDR(%): 13.12
Fungal_trans (PF04082)
357
523
551
577
Disorder Tendency
1.00
0.75
0.50
0.25
0.00
0
100
200
300
400
500
600
700
800
900
1000
1100
Amino Acid Position
Disordered
Ordered
C
Putative transcription factor SEF1 (Asp523-Glu551)
Asp523
Glu551
D
NADFUDRAFT_51267
(3',5'-cyclic-nucleotide phosphodiesterase, ODQ65997)
DSDR(%): 19.46
PDEase_II (PF02112)
123
203
260
420
Disorder Tendency
1.00
0.75
0.50
0.25
0.00
0
50
100
150
200
250
300
350
400
Amino Acid Position
Disordered
Ordered
E
3',5'-cyclic-nucleotide phosphodiesterase
Pro203
Ser260

Figure S18

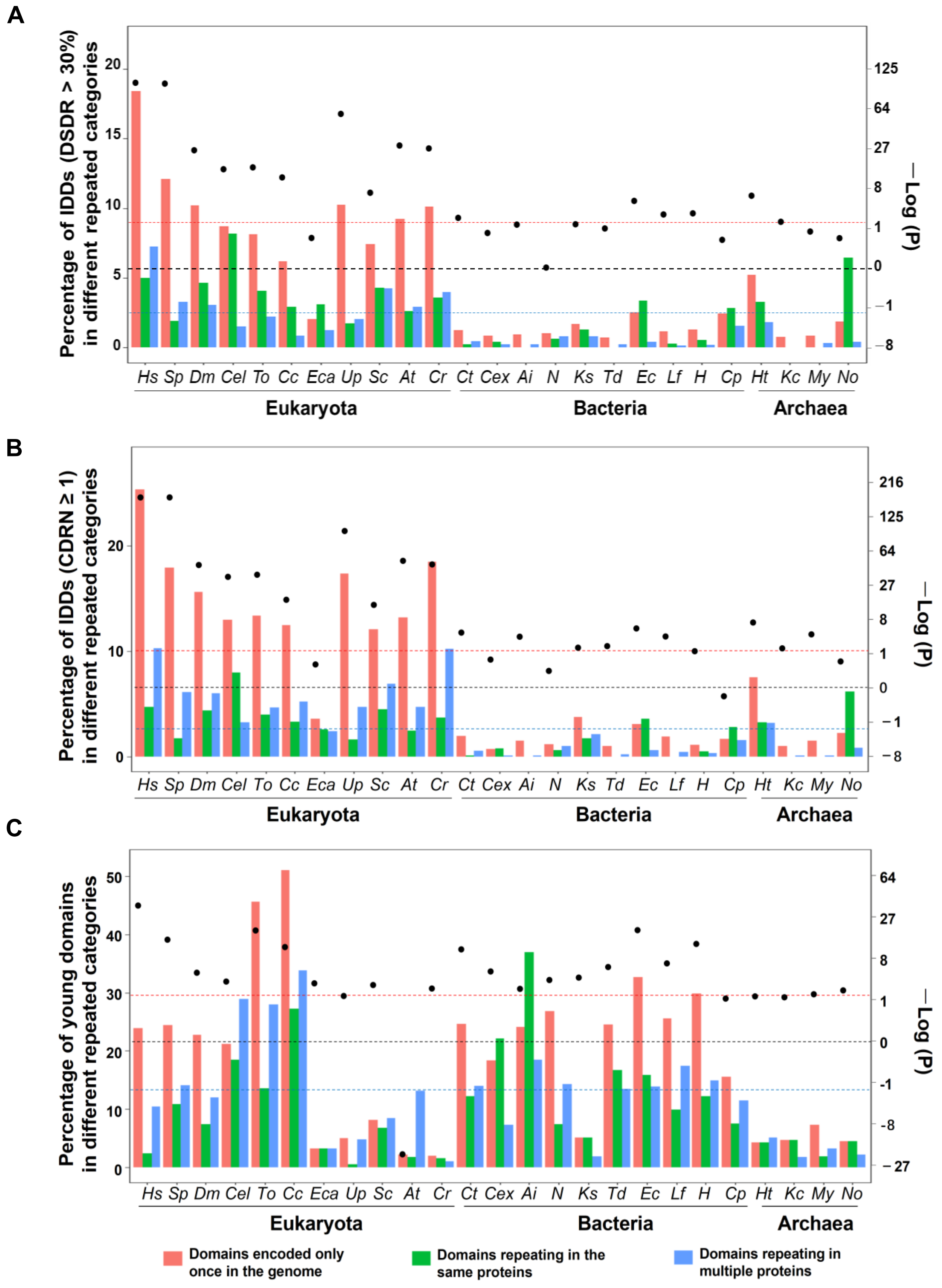

A
Percentage of IDDs (DSDR > 30%) in different repeated categories
— Log (P)
Hs Sp Dm Cel To Cc Eca Up Sc At Cr Ct Cex Ai N Ks Td Ec Lf H Cp Ht Kc My No
Eukaryota
Bacteria
Archaea
B
Percentage of IDDs (CDRN ≥ 1) in different repeated categories
C
Percentage of young domains in different repeated categories
Domains encoded only once in the genome
Domains repeating in the same proteins
Domains repeating in multiple proteins

Figure S19

A

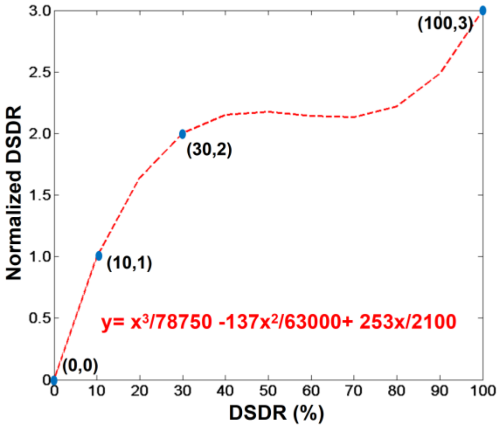

B

DSDR variation	Classification
0	No-variation
0-1	Low-variation
1-3	High-variation

C

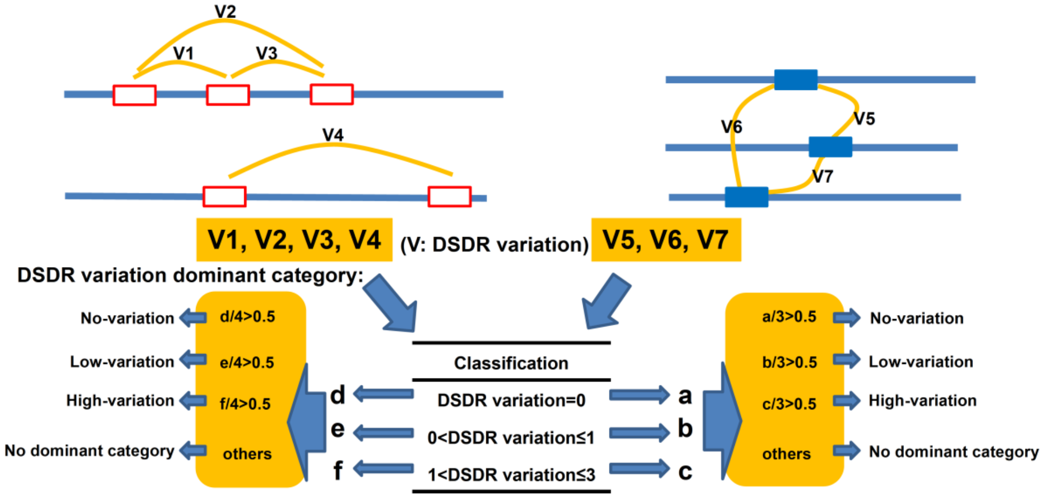

Figure S20

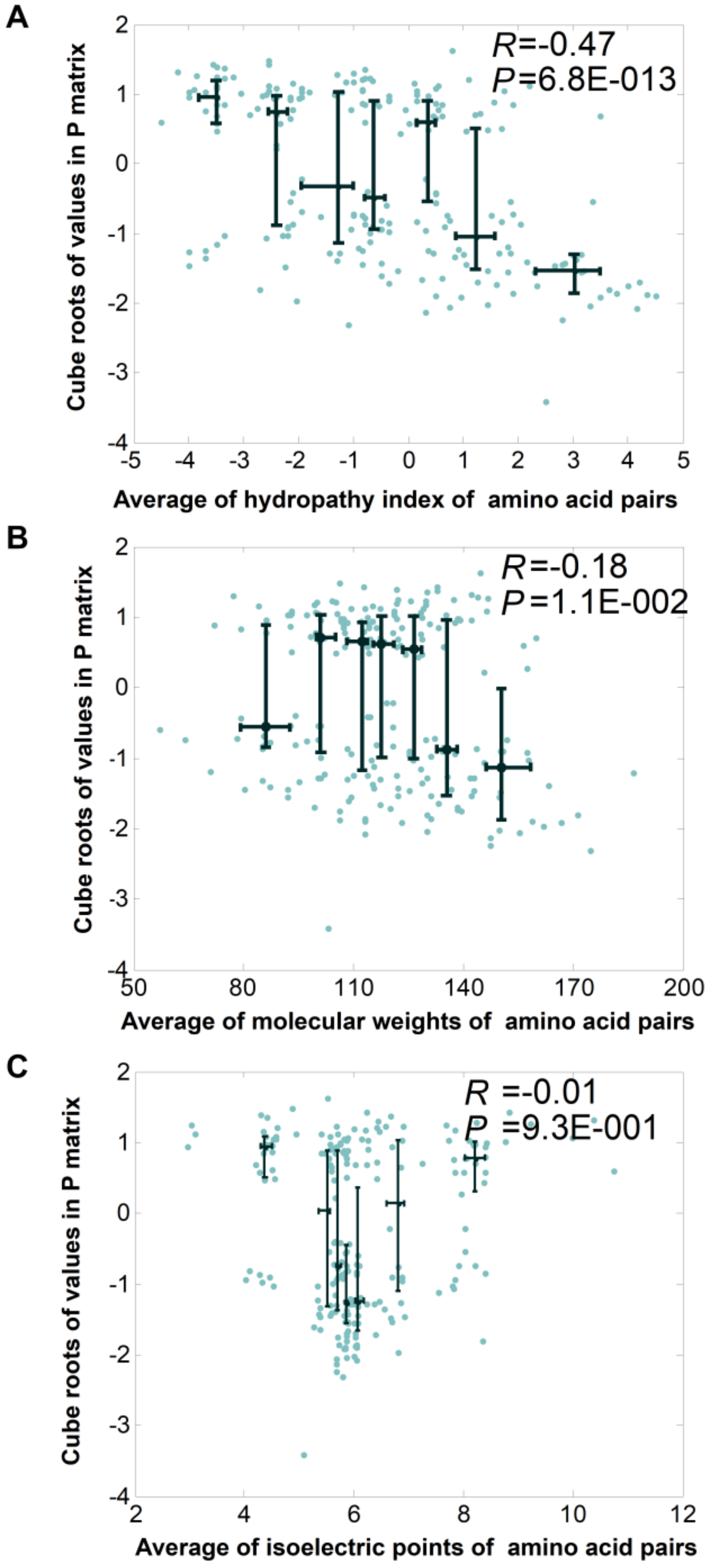
A
R=-0.47
P=6.8E-013
Cube roots of values in P matrix
Average of hydropathy index of amino acid pairs
B
R=-0.18
P=1.1E-002
Cube roots of values in P matrix
Average of molecular weights of amino acid pairs
C
R =-0.01
P =9.3E-001
Cube roots of values in P matrix
Average of isoelectric points of amino acid pairs

Figure S21

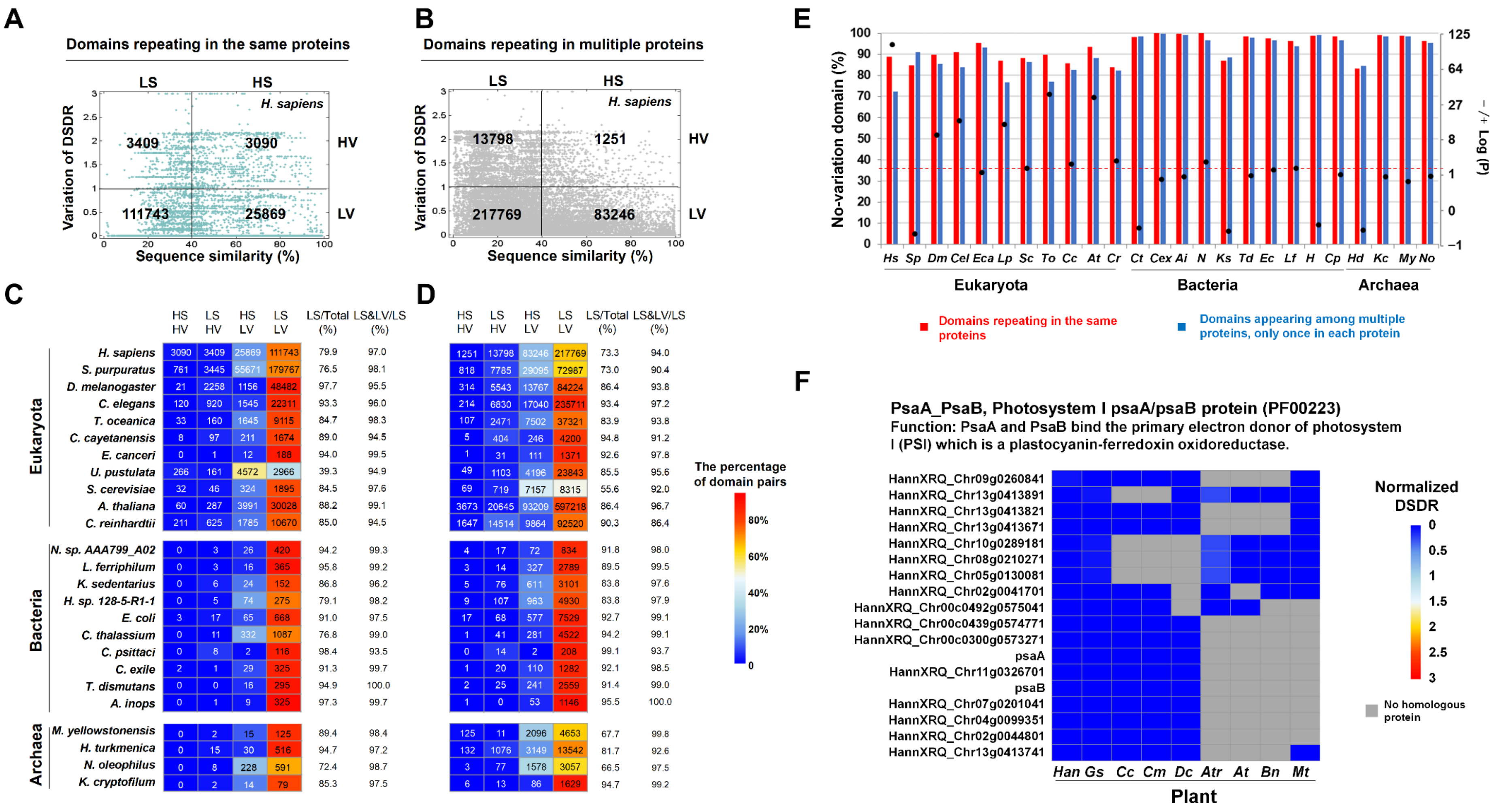
A
Domains repeating in the same proteins
LS
HS
H. sapiens
3409
3090
HV
111743
25869
LV
Variation of DSDR
Sequence similarity (%)
B
Domains repeating in mulitiple proteins
LS
HS
H. sapiens
13798
1251
HV
217769
83246
LV
Variation of DSDR
Sequence similarity (%)
C
HS HV | LS HV | HS LV | LS LV | LS/Total (%) | LS&LV/LS (%)
Eukaryota
H. sapiens 3090 3409 25869 111743 79.9 97.0
S. purpuratus 761 3445 55671 179767 76.5 98.1
D. melanogaster 21 2258 1156 48482 97.7 95.5
C. elegans 120 920 1545 22311 93.3 96.0
T. oceanica 33 160 1645 9115 84.7 98.3
C. cayetanensis 8 97 211 1674 89.0 94.5
E. canceri 0 1 12 188 94.0 99.5
U. pustulata 266 161 4572 2966 39.3 94.9
S. cerevisiae 32 46 324 1895 84.5 97.6
A. thaliana 60 287 3991 30028 88.2 99.1
C. reinhardtii 211 625 1785 10670 85.0 94.5
Bacteria
N. sp. AAA799_A02 0 3 26 420 94.2 99.3
L. ferriphilum 0 3 16 365 95.8 99.2
K. sedentarius 0 6 24 152 86.8 96.2
H. sp. 128-5-R1-1 0 5 74 275 79.1 98.2
E. coli 3 17 65 668 91.0 97.5
C. thalassium 0 11 332 1087 76.8 99.0
C. psittaci 0 8 2 116 98.4 93.5
C. exile 2 1 29 325 91.3 99.7
T. dismutans 0 0 16 295 94.9 100.0
A. inops 0 1 9 325 97.3 99.7
Archaea
M. yellowstonensis 0 2 15 125 89.4 98.4
H. turkmenica 0 15 30 516 94.7 97.2
N. oleophilus 0 8 228 591 72.4 98.7
K. cryptofilum 0 2 14 79 85.3 97.5
D
HS HV | LS HV | HS LV | LS LV | LS/Total (%) | LS&LV/LS (%)
1251 13798 83246 217769 73.3 94.0
818 7785 29095 72987 73.0 90.4
314 5543 13767 84224 86.4 93.8
214 6830 17040 235711 93.4 97.2
107 2471 7502 37321 83.9 93.8
5 404 246 4200 94.8 91.2
1 31 111 1371 92.6 97.8
49 1103 4196 23843 85.5 95.6
69 719 7157 8315 55.6 92.0
3673 20645 93209 597218 86.4 96.7
1647 14514 9864 92520 90.3 86.4
4 17 72 834 91.8 98.0
3 14 327 2789 89.5 99.5
5 76 611 3101 83.8 97.6
9 107 963 4930 83.8 97.9
17 68 577 7529 92.7 99.1
1 41 281 4522 94.2 99.1
0 14 2 208 99.1 93.7
1 20 110 1282 92.1 98.5
2 25 241 2559 91.4 99.0
1 0 53 1146 95.5 100.0
125 11 2096 4653 67.7 99.8
132 1076 3149 13542 81.7 92.6
3 77 1578 3057 66.5 97.5
6 13 86 1629 94.7 99.2
The percentage of domain pairs
80%
60%
40%
20%
0
E
No-variation domain (%)
– / + Log (P)
Hs Sp Dm Cel Eca Lp Sc To Cc At Cr Ct Cex Ai N Ks Td Ec Lf H Cp Hd Kc My No
Eukaryota
Bacteria
Archaea
Domains repeating in the same proteins
Domains appearing among multiple proteins, only once in each protein
F
PsaA_PsaB, Photosystem I psaA/psaB protein (PF00223)
Function: PsaA and PsaB bind the primary electron donor of photosystem I (PSI) which is a plastocyanin-ferredoxin oxidoreductase.
HannXRQ_Chr09g0260841
HannXRQ_Chr13g0413891
HannXRQ_Chr13g0413821
HannXRQ_Chr13g0413671
HannXRQ_Chr10g0289181
HannXRQ_Chr08g0210271
HannXRQ_Chr05g0130081
HannXRQ_Chr02g0041701
HannXRQ_Chr00c0492g0575041
HannXRQ_Chr00c0439g0574771
HannXRQ_Chr00c0300g0573271
psaA
HannXRQ_Chr11g0326701
psaB
HannXRQ_Chr07g0201041
HannXRQ_Chr04g0099351
HannXRQ_Chr02g0044801
HannXRQ_Chr13g0413741
Han Gs Cc Cm Dc Atr At Bn Mt
Plant
Normalized DSDR
No homologous protein

Figure S22

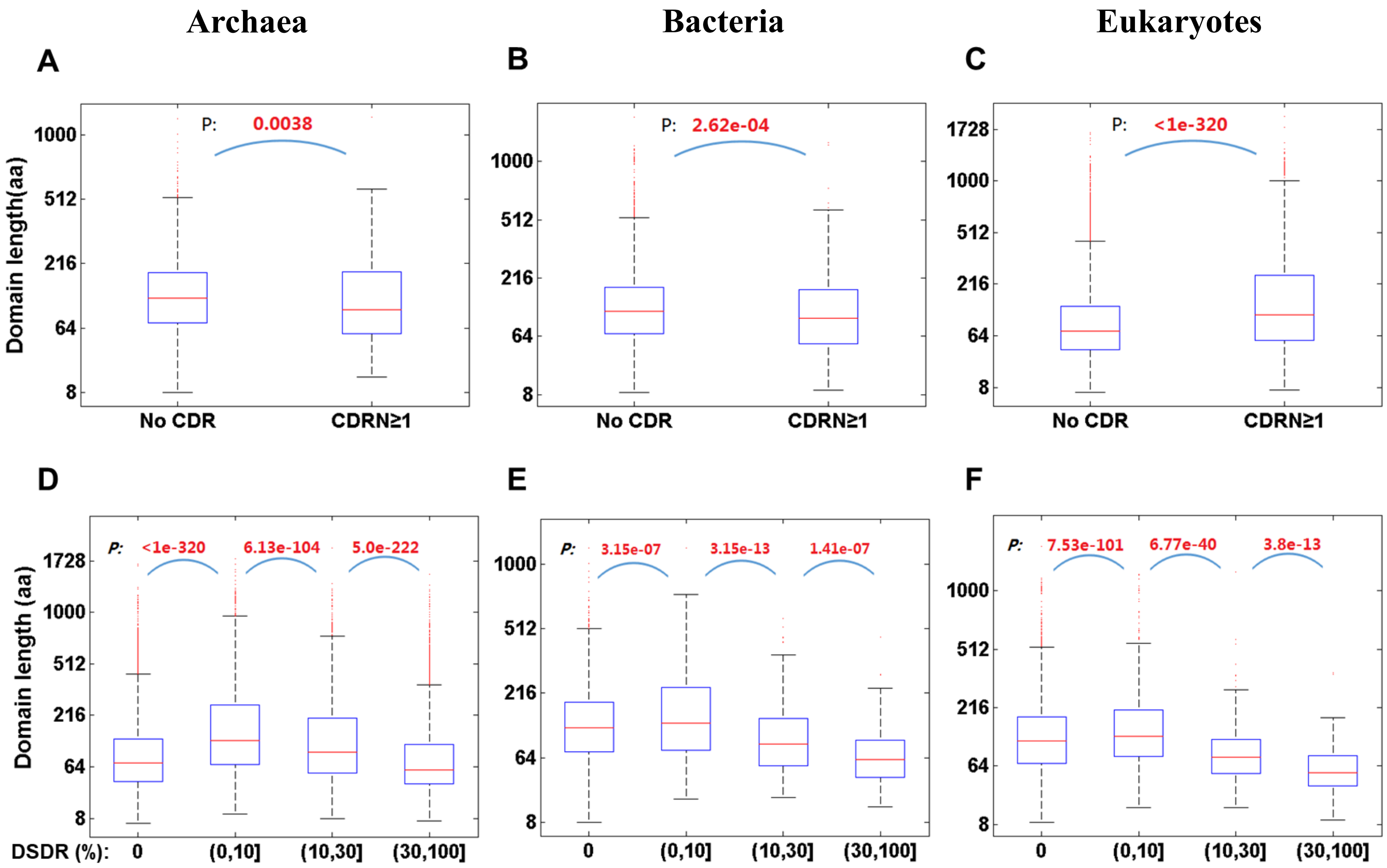
Archaea
Bacteria
Eukaryotes
A
B
C
Domain length(aa)
P: 0.0038
P: 2.62e-04
P: <1e-320
1000
512
216
64
8
1728
No CDR
CDRN≥1
D
E
F
Domain length (aa)
P: <1e-320 6.13e-104 5.0e-222
P: 3.15e-07 3.15e-13 1.41e-07
P: 7.53e-101 6.77e-40 3.8e-13
DSDR (%): 0 (0,10] (10,30] (30,100]
